# Design, Synthesis, and Docking Studies of New Torin2 Analogs as Potential ATR/mTOR Kinase Inhibitors †

**DOI:** 10.3390/molecules23050992

**Published:** 2018-04-24

**Authors:** Althaf Shaik, Rashmi Bhakuni, Sivapriya Kirubakaran

**Affiliations:** 1 Discipline of Chemistry, Indian Institute of Technology Gandhinagar, Palaj, Gandhinagar-382355, Gujarat, India; althaf.shaik@iitgn.ac.in; 2 Discipline of Biological Engineering, Indian Institute of Technology Gandhinagar, Palaj, Gandhinagar-382355, Gujarat, India; rashmi.bhakuni@iitgn.ac.in

**Keywords:** DNA damage and repair, ATR, ATM, mTOR, p-Chk1^S317^ (Ser 317), p70 S6K^T389^ (Thr 389), homology modeling, docking, molecular dynamic simulation

## Abstract

Targeting DNA damage and response (DDR) pathway has become an attractive approach in cancer therapy. The key mediators involved in this pathway are ataxia telangiectasia-mutated kinase (ATM) and ataxia telangiectasia-mutated, Rad3-related kinase (ATR). These kinases induce cell cycle arrest in response to chemo- and radio-therapy and facilitate DNA repair via their major downstream targets. Targeting ATP-binding site of these kinases is currently under study. Torin2 is a second generation ATP competitive mTOR kinase inhibitor (EC_50_ = 250 pmol/L) with better pharmacokinetic profile. Torin2 also exhibits potent biochemical and cellular activity against ATM (EC_50_ = 28 nmol/L) and ATR (EC_50_ = 35 nmol/L) kinases. In this study, eight new Torin2 analogs were designed and synthesized through multistep synthesis. All the synthesized compounds were characterized by NMR and mass analysis. The newly synthesized analogs were evaluated for their anti-cancer activity via CellTiter-Glo^®^ assay. Additionally, compounds **13** and **14** also showed significant inhibition for ATR and mTOR substrates, i.e., p-Chk1 Ser 317 and p70 S6K Thr 389, respectively. Compounds **13** and **14** displayed promising anti-cancer activity with HCT-116 cell lines in the preliminary study. Further, a comparative model of ATR kinase was generated using the SWISS-MODEL server and validated using PROCHECK, ProSA analysis. Synthesized compounds were docked into the ATP-binding site to understand the binding modes and for the rational design of new inhibitors.

## 1. Introduction

In a replicating mammalian cell, the DNA damage response and repair pathways are a complex network of signaling events involved in DNA damage repair, cell cycle checkpoints, and apoptosis which mediate the cellular response to endogenous or exogenous stresses. The DNA damage and repair pathways are dependent on signals transduced from the phosphoinositide 3-kinase (PI3K)-related protein kinases (PIKKs) family of serine/threonine kinases, including ataxia telangiectasia-mutated (ATM), ATM and Rad3-related (ATR), and DNA-dependent protein kinase (DNA-PK) [1,2].

In the context of cancer treatment, DNA damaging strategies such as chemotherapeutic agents and ionizing radiation are used to induce DNA damage and replication fork stalling. It leads to the formation of DNA breaks/lesions [3]. The signals emanating from DNA breaks/lesion lead to activation of ATR and ATM kinases to enforce cell cycle arrest [3,4]. ATR and ATM are generally assumed to respond to different types of DNA damage. ATR activation associated with UV light irradiation and single strand breaks (SSBs) leads to the phosphorylation of Chk1 kinase promoting S and G2 arrest [3,5]. Double strand breaks (DSBs—generated using ionizing radiation) activate ATM, which can phosphorylate Chk2 kinase leading to the activation of p53 followed by G1 arrest or apoptosis. Many studies show that these important mechanisms help cancer cells to survive these treatments [3]. Targeting these signaling pathways could potentially enhance the effectiveness of replication inhibitors. Recent studies suggest that ATRi’s (ATR inhibitors) kill cancer cells more effectively when combined with DNA damaging agents such as antimetabolites, alkylating agents, topoisomerase inhibitors, and platinating agents [1,6,7]. The possible advantage of this method would be the use of lower dosages of replication inhibitors, which would reduce the toxicity caused by these inhibitors.

**Figure 1:**
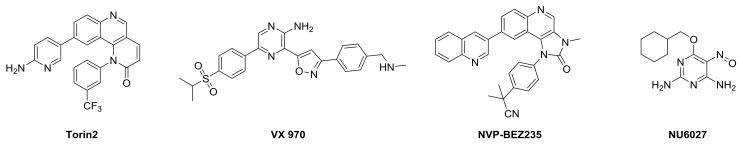
ATR inhibitors.

A number of potent and selective inhibitors have been developed for DNA-PK, mTOR, and ATR substrate Chk1 [8]. In contrast, the number of inhibitors specific to ATR is very small (Figure 1). However, there is growing interest in ATRi’s for cancer therapy [4]. Inhibitors like Torin2, VX970, NU6027, and NVP-BEZ235 have been found to have good potency toward ATR/ATM inhibition and other PI3K kinases in different cell lines [9].

Torin2 is a second generation ATP competitive mTOR inhibitor with IC_50_ of 0.25 nM, which also inhibits ATR and ATM kinase in a kinativ scanning. Torin2 exhibits potent cellular activity against ATR with EC_50_ of 35 nmoles/L (HCT-116 cell lines) [10]. Due to its selectivity towards the PIKK family, superior pharmacokinetic, and pharmacodynamics properties, we are interested in designing novel Torin2 analogs for ATR kinase inhibition.

Torin2 is a 9-(6-aminopyridin-3-yl)-1-(3(trifluoromethyl)phenyl)benzo[h][1,6]naphthyridin-2(1*H*)-one, derived from Torin1, has better half-life and bioavailability. Here, we have designed a few analogs of Torin2, in order to target PIKK family kinases like ATR, mTOR, ATM, and DNA-PK kinases, particularly ATR kinase inhibition. To achieve selectivity for ATR kinase, we have performed *in-silico* studies to validate our designed Torin2 analogs. Since there is no crystal structure of ATR kinase, we have generated an ATR kinase homology model.

The homology model for ATR kinase domain (2293–2567 amino acids) was built from SWISS Server using the crystal structure of mTOR kinase (PDBID: 4JSP), having a resolution of 3.3 Å. The generated model was validated using Ramachandran plot, PROCHECK, ProSA analysis, and MD simulation. Further, the designed Torin2 analogs were docked in to the ATR kinase homology model to understand the binding modes and for the rational design of new inhibitors.

## 2. Results

### 2.1. Synthesis

#### 2.1.1. Scheme 1

**Scheme 1:**
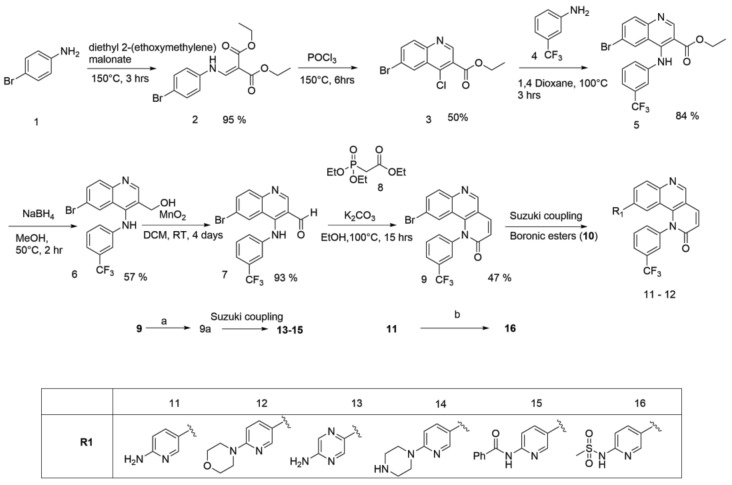
General procedure for the synthesis of Torin2 analogs 11–16: (a) boronic ester, CH_3_COOK, Pd(dppf)_2_Cl_2_, 1,4-dioxane, 100 °C, 12 h; (b) methanesulphonyl chloride, pyridine, DCM, 40 °C, 5 h.

Compound **1** was treated with dimethoxy methylene malonate to give **2**, which on aromatization followed by chlorination, in presence of POCl_3,_ gave an important intermediate **3** [11]. A nucleophilic substitution of **4** with **3** gave compound **5**, which, on reduction with NaBH_4_, gave **6**. Benzylic oxidation of **6** with MnO_2_ resulted in **7**, which, when followed by Horner-Wadsworth-Emmons olefination, produced compound **9**. Compound **9** on Suzuki coupling with corresponding boronic ester resulted in synthesis of Torin2 analogs (compound **11** and **12**) [8] Scheme 1.

#### 2.1.2. Scheme 2

**Scheme 2:**
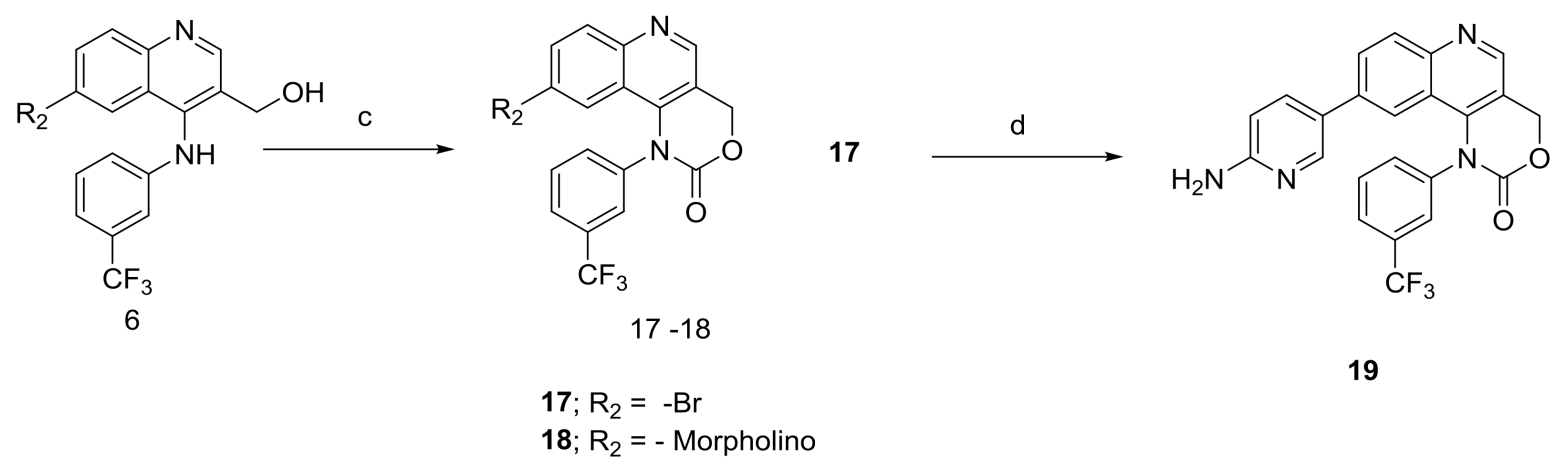
General procedure for the synthesis of Torin2 analogs **17**–**19**: (c) diphospgene, Et_3_N, 1,4-Dioxane, 6 h, room temperature; (d) boronic ester, Pd(Ph_3_P)_3_Cl_2_, K_2_CO_3_, 1,4-Dioxane, 100 °C, 12 h.

6-Bromo or morpholine-substituted 4-((3-(trifluoromethyl)phenyl)amino)quinolin-3-yl)methanol was treated with trichloromethyl carbonochloridate in presence of Et_3_N at room temperature to give compound **17** and **18**. Compound **18** on Suzuki coupling with corresponding boronic ester produced Torin2 analogs (compound **11** and **13**) Scheme 2.

### 2.2. Homology Modeling and Docking Studiess

#### 2.2.1. Homology Modeling

**Figure 2:**
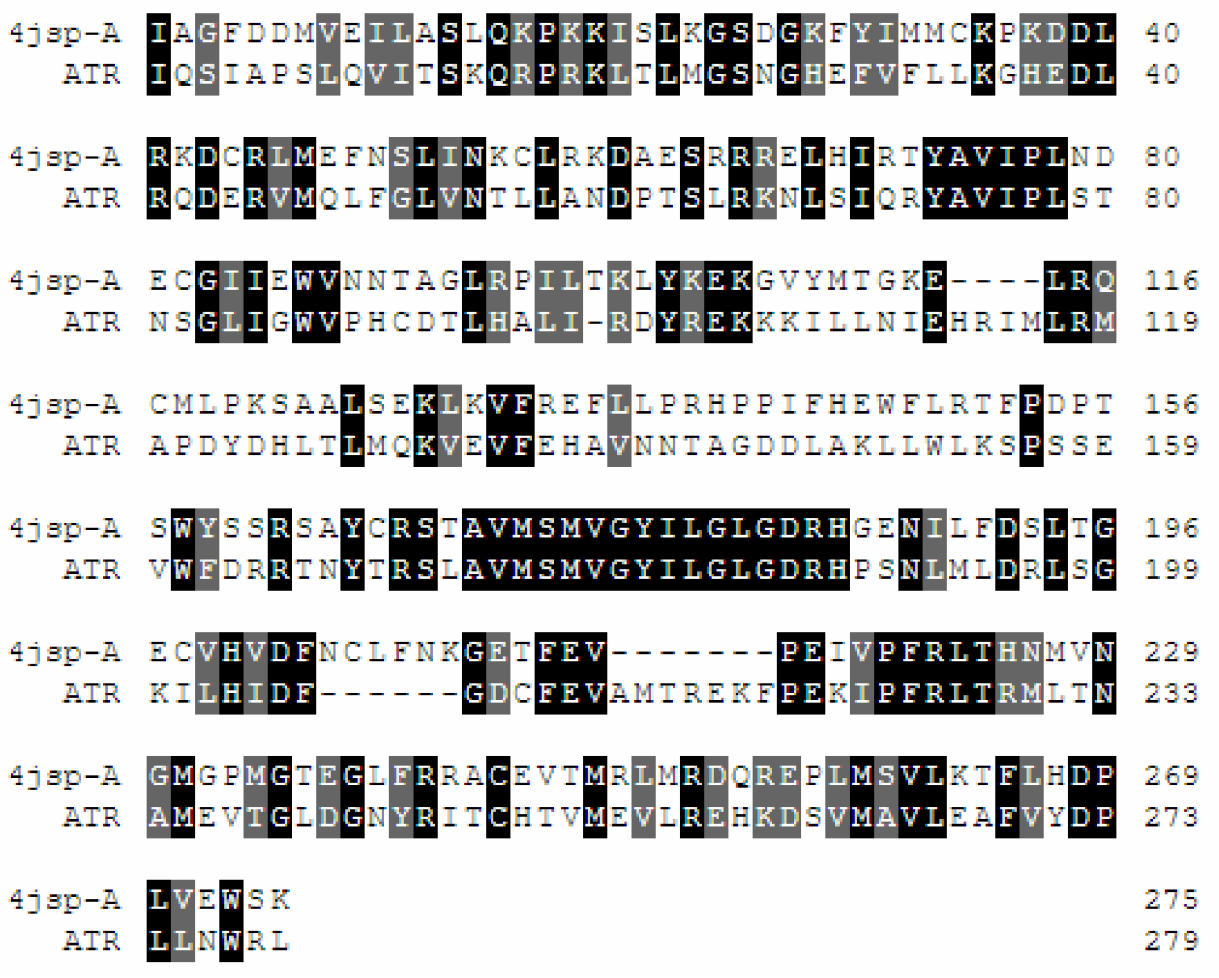
Pairwise alignment of 4JSP_A and ATR kinase.

**Figure 3:**
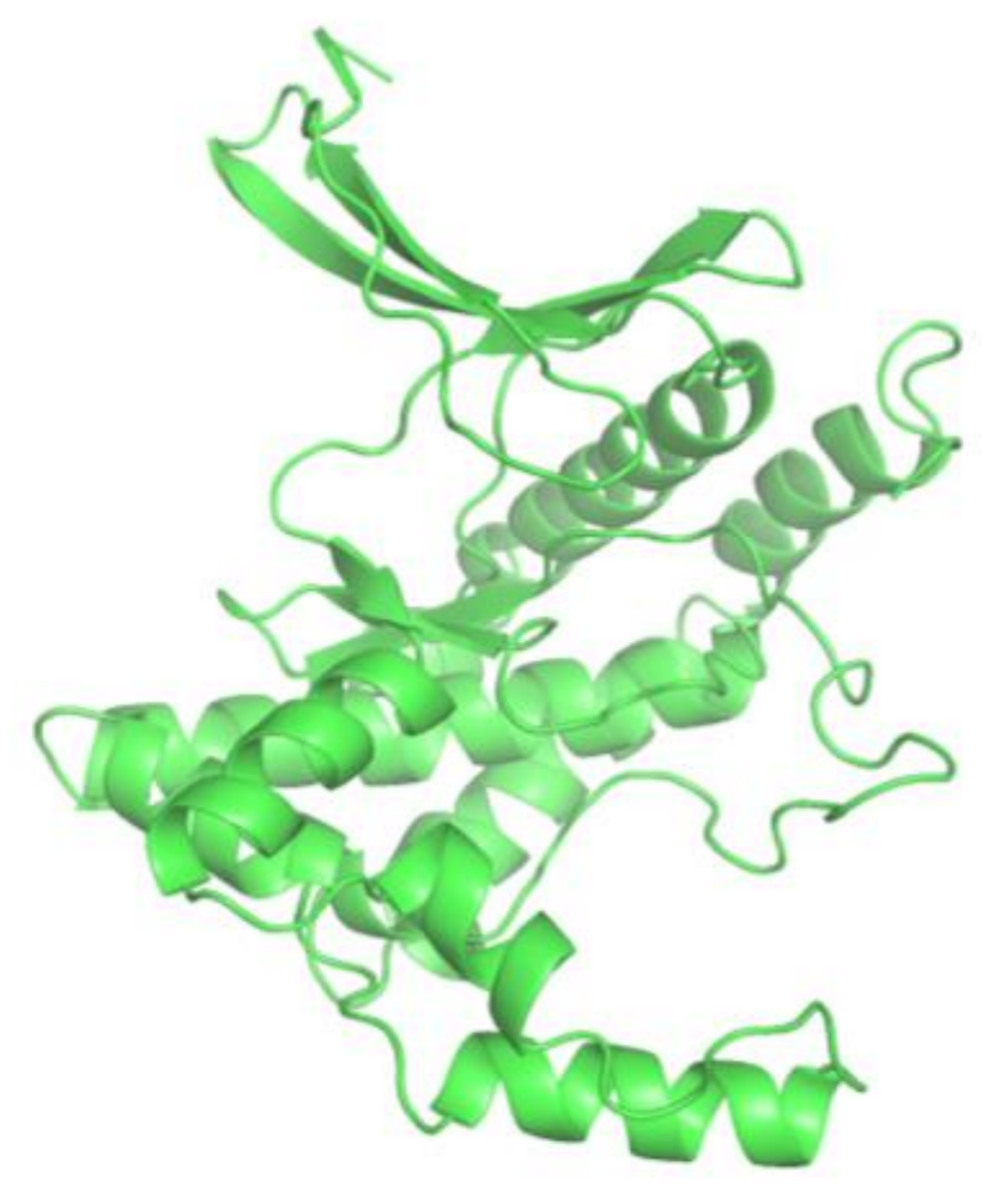
Constructed 3D model of ATR kinase.

The homology model of ATR (kinase domain) was constructed using SWISS-MODEL server [12]. SWISS-MODEL server is an automated comparative modeling server that is used for prediction of 3D structure of proteins [12]. ATR amino acid sequence was retrieved from NCBI (GenBank ID: CAA70298.1) database. It consists of 2644 amino acids, among which 2293–2567 belong to the kinase domain of ATR. This particular amino acid sequence was subjected to modeling using SWISS–MODEL server. The X-ray crystallographic structure of mTOR DeltaN–mLST8–ATP gamma S-Mg complex (4JSP) was selected as a template for modelling of ATR kinase domain. A resolution of 3.3 Å and 35.53% sequence identity with the query sequence made it a reliable template to build putative ATR kinase structure (Figure 2). The constructed 3D-structure of ATR kinase domain is a monomer, with N-terminal β-sheets and C-terminal α-helices (Figure 3). The comparative model of ATR kinase domain generated by SWISS-MODEL server was predicted to have a QMEAN of −3.23, suggesting that the modeled structure is of good quality since a QMEAN of −5 to 2 is considered to be a good quality structure (Figure 2). The generated 3D structure was deposited at the Protein Model Database (PMDB) and assigned the PMDB ID: PM0081472.

#### 2.2.2. Quality Assessment/Model Validation

The root mean square deviation (RMSD) was calculated by superposing 4JSP_A over generated model (i.e., the back bone atoms of alpha carbon) using SuperPose version 1.0 [13]. The Cα RMSD and the backbone RMSD deviations for the model and the template crystal structure were found to be 2.24 Å and 2.16 Å, respectively.

Further, the generated ATR kinase domain structure was evaluated using ProSA online. ProSA online server calculates the z-score, which specifies the quality of protein structure utilising energetics and geometric criteria. The z-score of the predicted ATR kinase model and template 4JSP_A were −6.88 and −14.10 (Figure 3). In general, the z-score should be below zero for no significant stressed or strained folds with high energies. Also, majority of peaks of the predicted model were below the peaks of 4JSP_A mTOR structure in the overlap energy profile graph, which manifests the quality of predicted ATR kinase model.

**Figure 4:**
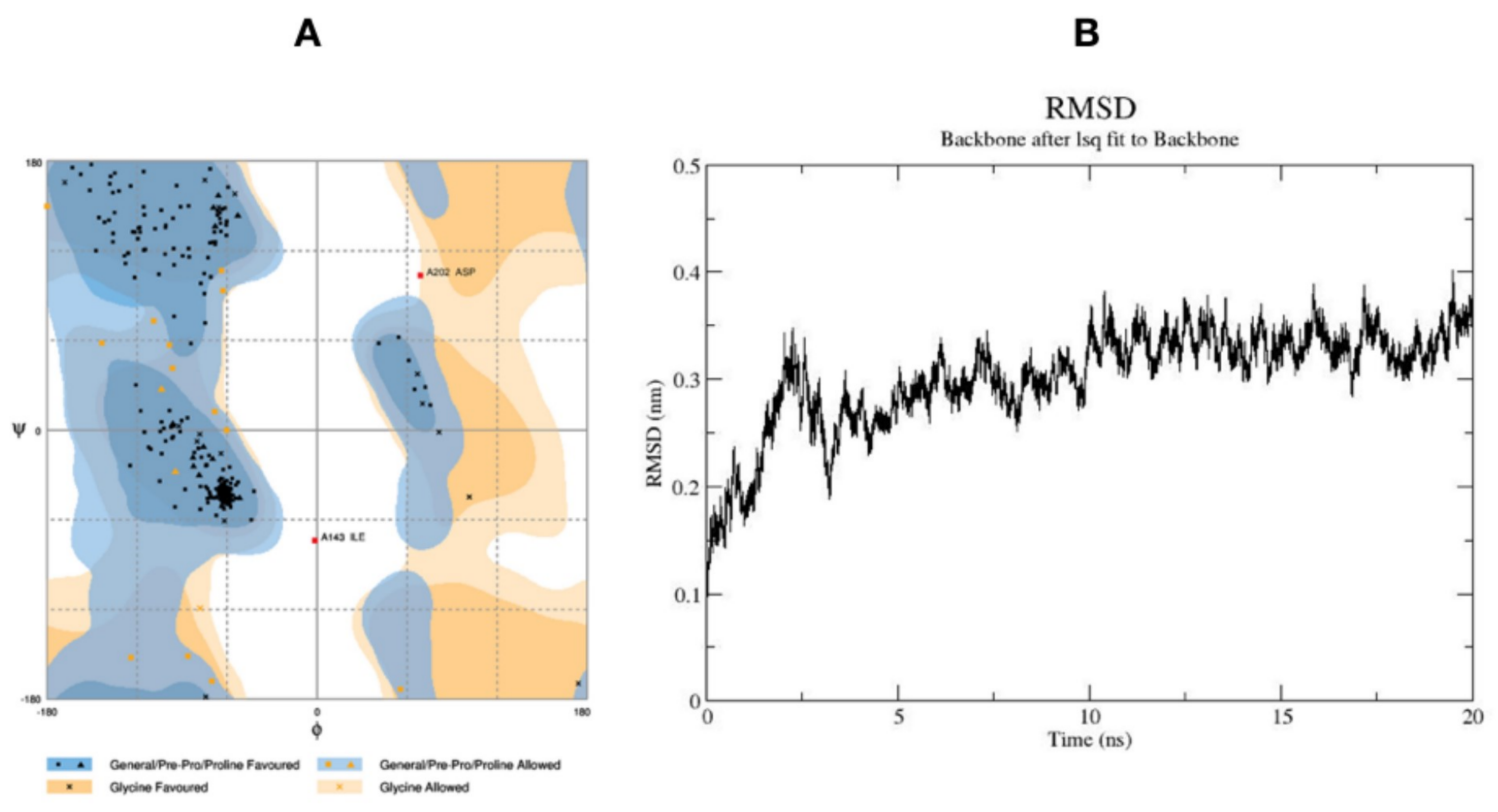
Ramachandran plot of generated ATR kinase model (**A**); and Molecular dynamic trajectory for 20 ns (**B**).

Ramachandran analysis was carried out using PROCHECK program to predict the stereo chemical quality and accuracy of generated model. Ramachandran plot analysis revealed 93.4% residues of modeled structure in the favoured region, 5.9% residues in the allowed region, and 0.7% residue in the disallowed region (Figure 4). The active site of ATR kinase was predicted by site map tool in Schrödinger and further confirmed by structural alignment onto to the 4JSP_A ATP binding pocket.

Based on the results obtained from PROCHECK, RMSD, and ProSA programme, the predicted ATR kinase model can be used further to study protein-protein interactions and molecular docking purpose.

#### 2.2.3. Molecular Dynamic Simulations and Docking Studies with Torin2

**Figure 5:**
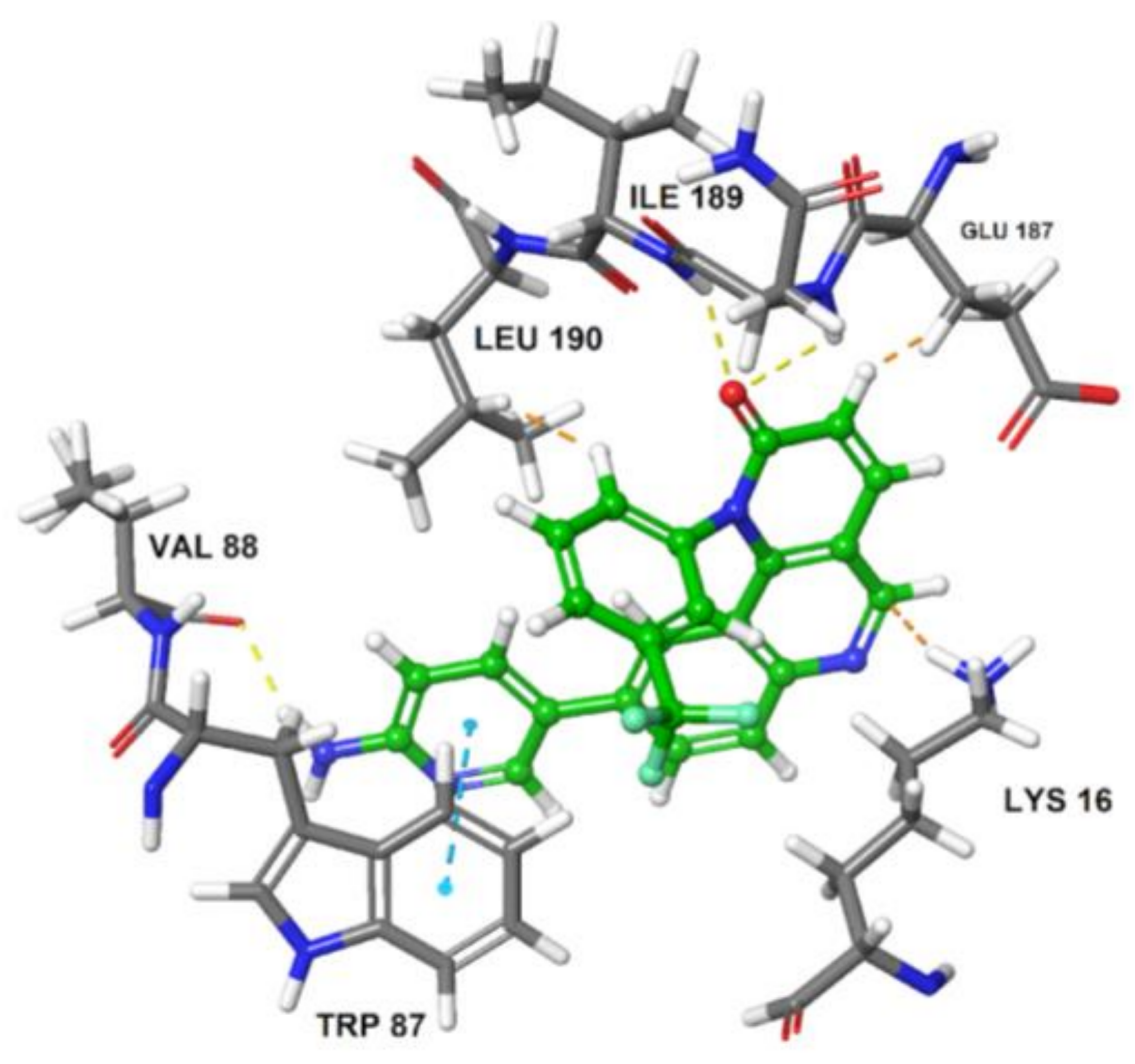
Binding pose of compound **11** (Torin2) with ATR kinase. The inhibitor is represented as ball and stick model (green color) and the key residues in the active site are presented as tube model (gray color).

Molecular dynamic simulation was performed to study the stability and dynamics of homology model and also to get the optimized structure of ATR kinase for docking studies. The RMSD plot of total energy and time dependence of back bone at 20 ns was generated (Figure 4). In the first 50 ps, RMSD values increased to 0.3 nm and then stabilized at 0.35 nm; further, there is no change in the conformational stability of the protein. The average structure of ATR kinase from MD simulation trajectory was extracted for performing docking studies using Maestro v11.2 (Maestro, Schrödinger, LLC, New York, NY, USA, 2017). Using Glide XP docking tool, known ATR inhibitor, Torin2 was docked into the ATP-binding pocket surrounded by residues LYS 16, ILE 85, TRP 87, VAL 88, THR 91, PRO 96, ILE 189, ASN 188, VAL 201, and ASP 202. The docking of Torin2 with ATR kinase gave XP glide score of −8.022 kcal/mol, in which Torin2-formed hydrogen bonds with LYS16 and ASN 89 residues in the active site. NH_2_ group of pyridine ring formed hydrogen bond with ASN 89 residue and another hydrogen bonding was observed between C=O of naphthyridone ring and LYS 16 residue. Also, phenyl moiety of quinolone ring formed π-π stacking interaction with TRP 87 (Figure 5).

#### 2.2.4. Docking of Designed Inhibitors and Binding Pose Analysis

Based on the above results, we have designed a few analogs of Torin2 to understand the effect of substitution pattern on docking score (Table 1). Compounds **13**, **15**, and **16** showed better to moderate docking score (−8.1, −8.2, and −6.5 kcal/mol) than Torin2 (Table 1). In case of **13,** substitution of pyridine ring of Torin2 with pyrazine amine ring lowered its docking score. Compound **15** and **16** are amide derivatives of Torin2, which were designed by replacing amino group with methyl sulphonamide group and benzamide moiety. Compounds **13**, **15**, and **16** form similar hydrogen bonding and π-π stacking with LYS 16, ASN 89, and TRP 87 residues of ATR. For compounds **12**, **17**, **18**, and **19**, we observed an increased docking score, i.e., −7.2 kcal/mol, −3.4 kcal/mol, −4.0 kcal/mol, and −4.3 kcal/mol when compared with Torin2. Compound **12** was designed to check the importance of NH_2_ hydrogen bonding with LYS 16 residue of ATR kinase, as well as to achieve better solubility. As expected, a lack of hydrogen bonding with LYS 16 residue was observed. Compounds **17**, **18**, and **19** were designed to explore the importance of naphthyridine ring of Torin2. Replacement of naphtyridine with 1,4-dihydro-2*H*-[1,3]oxazino ring resulted in compounds **17**, **18**, and **19**.

**Figure 6:**
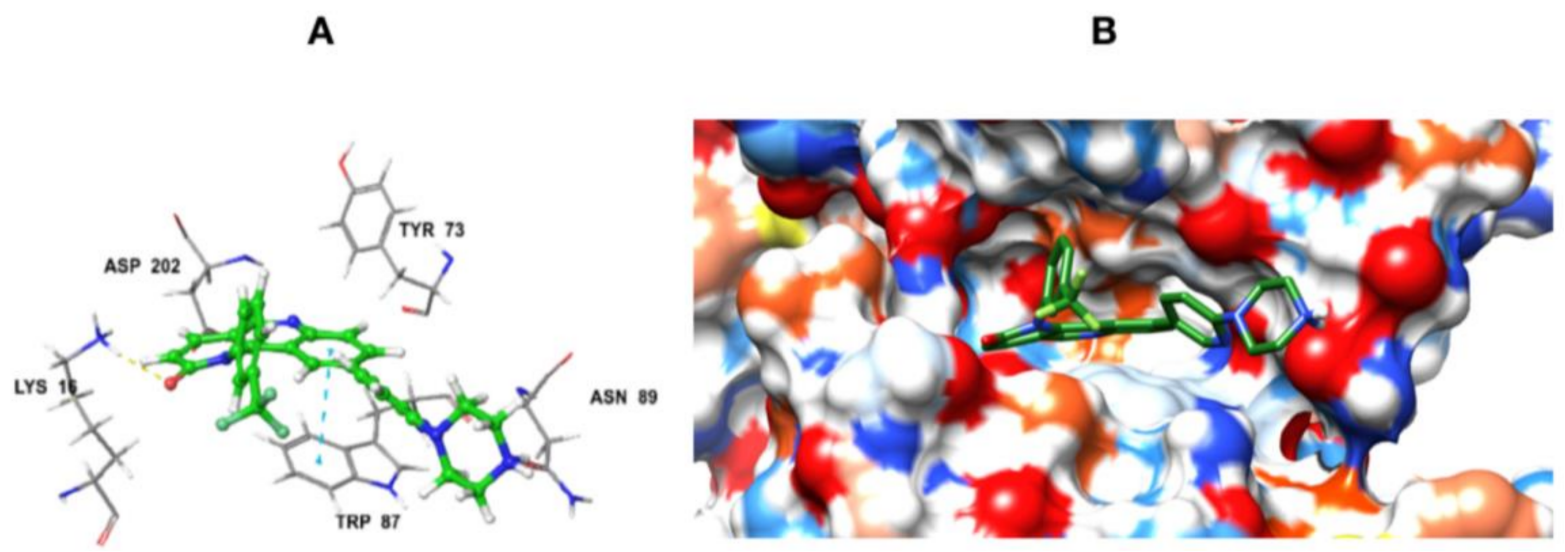
(**A**) Binding pose of compound **14** and its interaction with important residues of ATR kinase. The inhibitor is represented as ball and stick model (green color) and the key residues in the active site are presented as tube model (gray color); (**B**) Electrostatic potential surface representation of ATR kinase binding cavity (blue color: positive potential, red color: negative potential, green color: inhibitor tube model).

Addition of 1,4-dihydro-2*H*-[1,3]oxazino introduced slight conformational changes but still managed π-π and hydrogen bonding with LYS 16 and TRP 87 residues. The increased docking score was observed due to loss of hydrogen bonding with ASN89 residue. Compound **14** was designed to extend the hydrogen bonding with nearby residue, ASN 89, of ATR kinase, in which a piperzyl ring was added to 2nd position of pyridine ring of Torin2. NH of piperzyl ring was able to form strong hydrogen bonding with ASN 89 residue and showed a docking score of −7.0 kcal/mol (Figure 6).

Particularly, NH_2_ or NH group of pyridine ring and C=O of pyridinone ring of ATRi’s with Torin2 scaffold are needed for its anticancer activity.

### 2.3. Cell Viability Assay

Human HCT-116 cells were grown in DMEM/10% FBS/1% Pen-Strep medium at 37 °C in a humidified incubator with 5% CO_2_. For the cell viability assay, cells were plated into 96-well plates at a count of 2000 cells per well in 198 μL medium, incubated for 24 h, and then treated with increasing concentrations of compound, respectively. After 72 h of compound treatment, cell viability was determined using CellTiter-Glo^®^ Luminescent assay (Promega, Madison, WI, USA). Luminescence was measured by Envision Hybrid and modular multimode reader. Data was analysed by GraphPad Prism 6 software (GraphPad Software Inc., La Jolla, CA, USA) to get GI_50_ of each compound.

### 2.4. Immunoblot Assay

HCT-116 cells were seeded in 6-well plates at a count of 0.5 × 10^6^ cells per well and incubated overnight in a humidified CO_2_ incubator maintained at 37 °C. For ATR assay, cells were exposed to 50 mJ/cm^2^ of UV radiation energy (using UVP cross linker) after an hour of pre-treatment with appropriate compounds. Culture media was saved before UV treatment and added back to the cells after UV treatment. After another 1-h incubation, cells were rinsed with ice-cold PBS and lysed in ice-cold cell extraction buffer. The soluble fractions of cell lysate were isolated by centrifugation at 13,000 rpm for 10 min at 4 °C. Following that, concentration of the protein was normalized by Bradford assay. Cell lysates were then subjected to SDS-PAGE and immunoblotting.

## 3. Discussion

In the present investigation, we have used computational approach to model ATR kinase structure using SWISS-MODEL server and validated it using PROCHECK, ProSA, and MD simulation. Ramachandran plot analysis of generated model showed 93.4%, 5.9%, and 0.7% residues in favored, allowed, and disfavored regions. Generated ATR kinase model was utilized to design novel Torin2 analogs for effective inhibition of ATR kinase. Molecular docking studies using Glide XP tool were utilized to rationalize the designed inhibitors. Inhibitors with best docking score and strong interactions with ATR kinase were synthesized (multi-step synthesis) and characterized by NMR and mass spectrometry.

**Figure 7:**
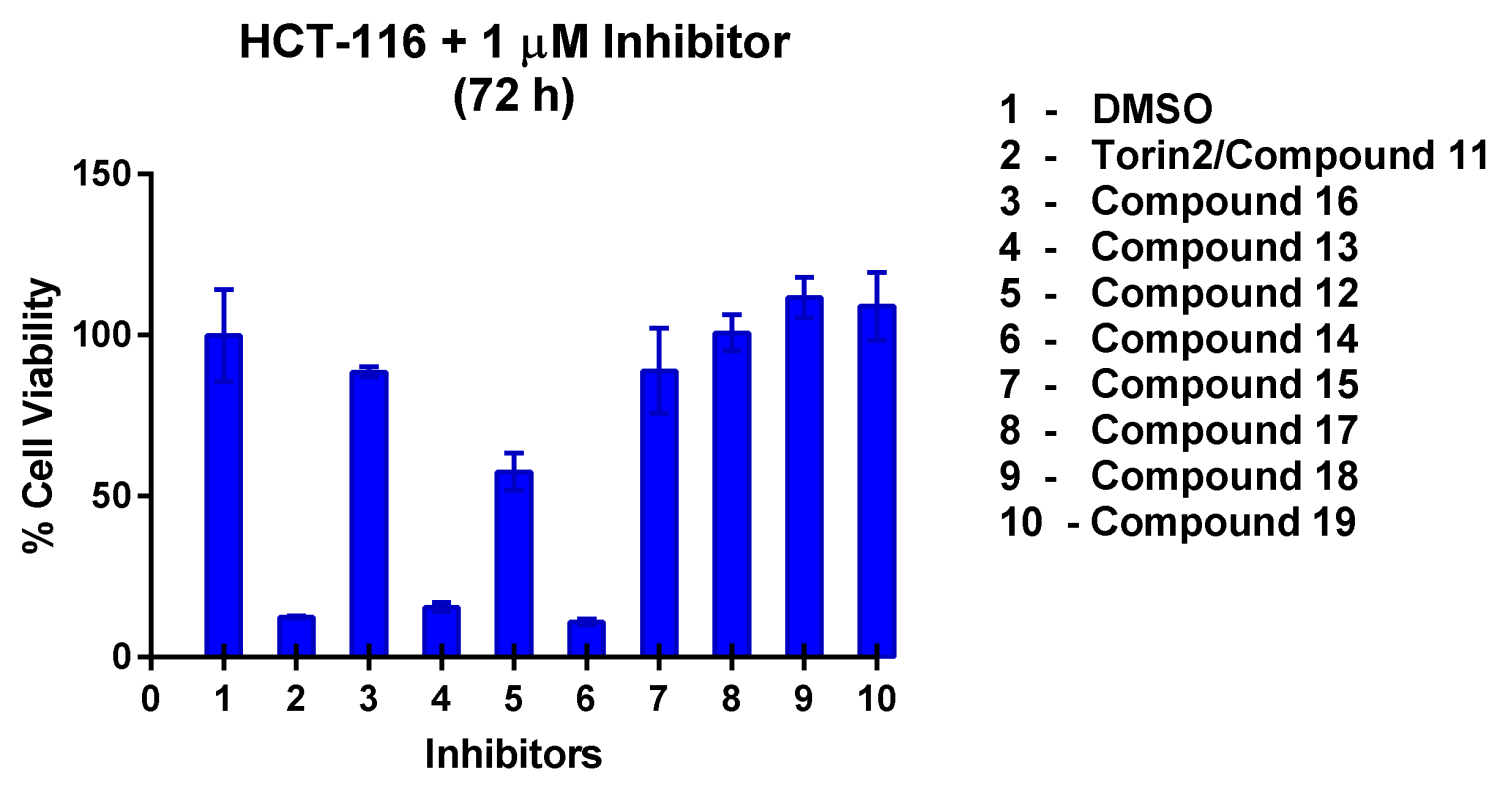
HCT-116 cell line was treated with synthesized compounds at 1 µM for 72 h. Compounds **13** and **14** showed good potency similar to Torin2/compound **11**.

The growth inhibition profile of all the synthesized compounds was performed against HCT-116 cell lines at 1 µM concentration (Figure 7). Among the designed compounds, **13** and **14** showed strong inhibition similar to compound **11** (Torin2). Two novel potent compound inhibitors, i.e., **13** and **14**, were selected from the initial screening based cell viability assay and used to sensitize colon cancer cell line. We performed another cell viability assay across a dose range (0–1000 nM) for both the compounds in colon cancer cell line. With an incubation time of up to 72 h, compound **14** was found to be more toxic than **13** with a GI_50_ of 57 nM (Figure S38). Compound **13** also inhibited viability of colon cancer cells, with a GI_50_ of 138 nM (Figure S38). We confirmed that both **13** and **14** helped in sensitization of HCT-116 cell line.

Based on the above mentioned results, to confirm if the compounds inhibit ATR and mTOR signaling in colon cancer cell line, an immunoblot assay was performed. UV-irradiation was used as a part of combinatorial treatment to induce DNA damage. We assessed phosphorylation of Chk1 (p-Chk1^S317^) and p70 S6 kinase (p70 S6K^T389^) via immunoblotting of treated HCT 116 cells. p-Chk1^S317^ and p70 S6K^T389^ are downstream targets of ATR and mTOR kinase, respectively. As expected, Torin 2 analogs, compounds **13** and **14**, inhibit ATR and mTOR kinase phosphorylation. It was observed that 250 nM of **14** treatment inhibited p-Chk1 (Ser 317) after treatment with UV radiation (Figure S40). Interestingly, phosphorylation of p70 S6 kinase (Thr 389) was observed to be inhibited by both the compounds at 50 nM for **14** and 250 nM for **13** (Figures S40 and S41). Importantly, we confirmed that compound **14** and **13** inhibited phosphorylation of ATR and mTOR substrates when used as a part of combinatorial treatment, but the inhibition of compound **13** was found to be more towards mTOR rather than ATR. mTOR kinase inhibitors have already been well reported in literature [14], but there are no specific ATR inhibitor/s known till date [5]. We believe the ability of compounds **13** and **14** to inhibit ATR and mTOR kinase pathway can prove useful in cases in which mTOR inhibition alone is ineffective [10]. Also, more SAR studies are under progress to search for specific ATR inhibitors.

## 4. Materials and Methods 

The starting materials, solvents, and reagents used for synthesis were obtained from Sigma Aldrich (Darmstadt, Germany), Spectrochem, Merck (Darmstadt, Germany), and S. D. Fine chemicals Ltd. (Mumbai, India). Solvents utilized in the synthesis were dried thoroughly using reported procedures. ^1^H- and ^13^C-NMR spectra were recorded on Bruker AV500 (500 MHz) spectrophotometer (Bruker EPR Technology, Billerica, MA, USA) using tetramethylsilane (TMS) as an internal standard with chloroform-d (CDCl3) and dimethyl sulfoxide (d6) as the solvent. Mass spectral data was obtained using ESI-QToF (Waters-Synapt G2S) mass spectrometer (Waters, Milford, MA, USA).

### 4.1. Chemistry 

#### 4.1.1. Synthesis of Torin2 Analogs (Compounds **11**–**12**)

To a solution of compound **9** (0.1 g, 0.23 mmol) in 1,4-dioxane (2 mL) at room temperature in a sealed tube was added corresponding pyridyl boronic ester (0.17 g, 0.59 mmol), Pd(Ph_3_P)_3_Cl_2_ (0.023 mmol), and K_2_CO_3_ (1N, 0.69 mmol) under N_2_ atmosphere. The reaction mixture was degassed for 5–10 min. After degassing, the reaction mixture was heated at 100 °C for 15 h. The reaction mixture was then cooled to room temperature and directly concentrated under high vacuum. To the resulting residue, ice-cold water was added, and the mixture was extracted twice with ethyl acetate. The combined organic layers extract was washed with brine and dried over anhydrous Na_2_SO_4_. Solvent was evaporated under reduced pressure. The resulting mixture was purified by column chromatography (the compound was eluted at 5% MeOH: DCM) to afford compound **11**–**12** as yellow solid.


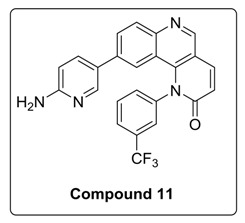


^1^H-NMR (500 MHz, DMSO-*d*_6_) δ 9.11 (s, 1H), 8.33 (d, *J* = 9.4 Hz, 1H), 8.13 (s, 1H), 8.06–8.01 (m, 2H), 7.94 (d, *J* = 8.7 Hz, 1H), 7.90 (t, *J* = 7.9 Hz, 1H), 7.81 (d, *J* = 8.1 Hz, 1H), 7.74 (d, *J* = 2.6 Hz, 1H), 7.05 (d, *J* = 8.6 Hz, 1H), 6.93 (d, *J* = 9.0 Hz, 2H), 6.40 (d, *J* = 8.7 Hz, 1H), 6.21 (s, 2H), 5.76 (s, 1H).

^13^C-NMR (126 MHz, DMSO-d6) δ 163.08, 159.094, 150.79, 148.24, 146.20, 142.13, 141.99, 140.79, 135.49, 133.92, 131.36, 128.26, 127.06, 126.46, 122.85, 122.12, 120.78, 117.94, 114.02, 108.15.

^19^F-NMR (470 MHz, DMSO-d6) δ −60.98.

MS (ESI): Exact mass for C_28_H_21_F_3_N_4_O_2_ = 432.12. Found (M + 1) = *m/z* 433.06


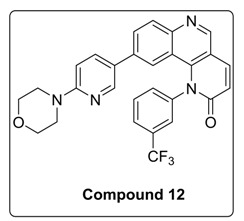


^1^H-NMR (500 MHz, Chloroform-*d*): δ 8.97 (s, 1H), 8.15 (d, *J* = 8.7 Hz, 1H), 8.08 (d, *J* = 2.5 Hz, 1H), 8.01 (d, *J* = 9.5 Hz, 1H), 7.90 (d, *J* = 8.0 Hz, 1H), 7.83 (dd, *J* = 8.7, 2.0 Hz, 1H), 7.77 (d, *J* = 7.9 Hz, 2H), 7.56 (d, *J* = 8.0 Hz, 1H), 7.06 (dd, *J* = 8.9, 2.6 Hz, 1H), 6.99 (d, *J* = 2.0 Hz, 1H), 6.95 (d, *J* = 9.4 Hz, 1H), 6.56 (d, *J* = 8.8 Hz, 1H), 3.85 (t, *J* = 4.8 Hz, 4H), 3.56 (t, *J* = 4.8 Hz, 4H).

^13^C-NMR (126 MHz, CDCl_3_) δ 163.17, 158.89, 149.96, 148.62, 146.32, 142.14, 141.26, 139.66, 135.80, 135.69, 132.48, 131.47, 131.12, 128.47, 126.27, 126.20, 124.88, 122.27, 121.64, 117.83, 113.73, 106.29, 66.71, 45.48.

^19^F-NMR (470 MHz Chloroform-*d*): −62.51.

MS (ESI): Exact mass for C_28_H_21_F_3_N_4_O_2_ = 502.16. Found (M + 1) = *m/z* 503.15.

#### 4.1.2. Synthesis of 9-(5-Aminopyrazin-2-yl)-1-(3-(trifluoromethyl)phenyl)benzo[h][1,6]naphtha yridin-2(1*H*)-one (Compound **13**)


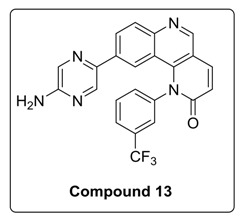


To a solution of compound **9a** (0.1 g, 0.21 mmol) in DMF (2 mL) at room temperature in a sealed tube was added 5-bromopyrazin-2-amine (0.055 g, 0.32 mmol), Pd(dppf)_2_ Cl_2_ (0.l eq), and K_3_PO_4_ in water (1.5 mmol) under N_2_ atmosphere. The reaction mixture was degassed for 5–10 min. After degasing, the reaction mixture was heated at 130 °C for 12 h and then cooled to room temperature The resulting mixture was directly concentrated under high vacuum. To the resulting residue, ice-cold water was added, and the mixture was extracted twice with ethyl acetate. The combined organic layers extract was washed with brine and dried over anhydrous Na_2_SO_4_. Solvent was evaporated under reduced pressure. The resulting mixture was purified by column chromatography (the compound was eluted at 5% MeOH: DCM) to afford compound **13** as yellow solid (24%).

^1^H-NMR (500 MHz, DMSO-d6) δ 9.12 (s, 1H), 8.33 (d, *J* = 9.4 Hz, 1H), 8.24 (dd, *J* = 8.7, 1.8 Hz, 1H), 8.12 (d, *J* = 2.2 Hz, 1H), 8.06 (d, *J* = 8.7 Hz, 1H), 7.99 (d, *J* = 7.9 Hz, 1H), 7.93 (d, *J* = 1.4 Hz, 1H), 7.85 (t, *J* = 7.9 Hz, 1H), 7.81 (d, *J* = 1.4 Hz, 1H), 7.73 (d, *J* = 8.0 Hz, 1H), 7.42 (d, *J* = 1.7 Hz, 1H), 6.94 (d, *J* = 9.5 Hz, 1H), 6.70 (s, 2H).

^13^C-NMR (300 MHz, DMSO-d6) δ 163.09, 155.52, 151.07, 148.88, 142.43, 141.75, 140.75, 139.10, 137.99, 134.43, 133.42, 131.93, 131.09, 127.09, 126.96, 126.30, 122.11, 120.94, 117.79, 113.99. 

^19^F-NMR (470 MHz, DMSO-d6) δ −61.01.

MS (ESI): Exact mass for C_23_H_14_F_3_N_5_O = 433.11. Found (M + 1) = *m/z* 434.12

#### 4.1.3. Synthesis of 9-(6-(Piperazin-1-yl)pyridin-3-yl)-1-(3-(trifluoromethyl)phenyl)benzo[h][1,6]naphthyridin-2(1H)-one (compound **14**)


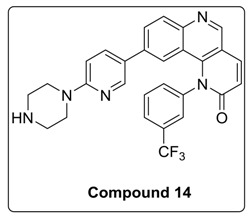


To a solution of compound **9a** (0.1 g, 0.21 mmol) in DMF (2 mL) at room temperature in a sealed tube was added piperzine substituted pyridine (0.31 mmol), Pd(dppf)_2_ Cl_2_ (0.l eq) and K_3_PO_4_ in water (1.5 mmol) under N_2_ atmosphere. The reaction mixture was degassed for 5–10 min. After degassing, the reaction mixture was heated at 130 °C for 12 h and then cooled to room temperature. The resulting mixture was directly concentrated under high vacuum. To the resulting residue, ice-cold water was added, and the mixture was extracted twice with ethyl acetate. The combined organic layers extract was washed with brine and dried over anhydrous Na_2_SO_4_. Solvent was evaporated under reduced pressure. The resulting mixture was purified by column chromatography (the compound was eluted at 5% MeOH: DCM) to afford compound **14** as yellow solid (35%).

^1^H-NMR (500 MHz, Chloroform-*d*) δ 8.96 (d, *J* = 2.0 Hz, 1H), 8.15 (d, *J* = 8.7 Hz, 1H), 8.08 (s, 1H), 8.01 (dd, *J* = 9.5, 2.0 Hz, 1H), 7.90 (d, *J* = 8.0 Hz, 1H), 7.83 (d, *J* = 8.6 Hz, 1H), 7.80–7.72 (m, 2H), 7.56 (d, *J* = 7.9 Hz, 1H), 7.03 (d, *J* = 8.9 Hz, 1H), 6.99 (d, *J* = 2.0 Hz, 1H), 6.95 (d, *J* = 9.4 Hz, 1H), 6.56 (d, *J* = 9.0 Hz, 1H), 3.59 (t, *J* = 5.0 Hz, 4H), 3.03 (t, *J* = 5.0 Hz, 4H).

^13^C-NMR (126 MHz, Chloroform-*d*) δ 163.22, 158.93, 149.90, 148.56, 146.31, 142.13, 141.24, 139.70, 135.64, 132.48, 131.42, 131.15, 128.48, 126.25, 124.36, 122.25, 121.53, 117.85, 113.72, 106.39, 46.01, 45.72.

^19^F-NMR (470 MHz, Chloroform-*d*) δ −62.50.

MS (ESI): Exact mass for C_28_H_22_F_3_N_5_O = 501.17. Found (M + 1) = *m/z* 502.21.

#### 4.1.4. Synthesis of *N*-(5-(2-Oxo-1-(3-(trifluoromethyl)phenyl)-1,2-dihydrobenzo[h][1,6]naphthyridin-9-yl)pyridin-2-yl)benzamide (compound **15**)


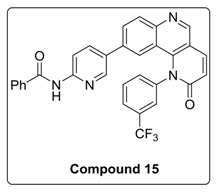


To a solution of compound **9a** (0.1 g, 0.21 mmol) in DMF (2 mL) at room temperature in a sealed tube was added *N*-(5-bromopyridin-2-yl)benzamide (0.32 mmol), Pd(dppf)_2_ Cl_2_ (0.l eq) and K_3_PO_4_ (1.5 mmol) under N_2_ atmosphere. The reaction mixture was degassed for 5–10 min. After degassing, the reaction mixture was heated at 130 °C for 12 h and then cooled to room temperature. The resulting mixture was directly concentrated under high vacuum. To the resulting residue, ice-cold water was added, and the mixture was extracted twice with ethyl acetate. The combined organic layers extract was washed with brine and dried over anhydrous Na_2_SO_4_. Solvent was evaporated under reduced pressure. The resulting mixture purified by column chromatography (the compound was eluted at 5% MeOH: DCM) to afford compound **15** as white solid (42%).

^1^H-NMR (500 MHz, Chloroform-*d*) δ 9.02 (s, 1H), 8.62 (s, 1H), 8.39 (d, *J* = 8.7 Hz, 1H), 8.22 (d, *J* = 8.7 Hz, 1H), 8.08–8.00 (m, 2H), 7.96 (d, *J* = 7.6 Hz, 3H), 7.88 (d, *J* = 8.5 Hz, 1H), 7.82 (t, *J* = 7.9 Hz, 1H), 7.71 (s, 1H), 7.66 (d, *J* = 8.0 Hz, 1H), 7.60 (d, *J* = 7.5 Hz, 1H), 7.55 (d, *J* = 7.5 Hz, 2H), 7.43 (d, *J* = 8.6 Hz, 1H), 7.07 (s, 1H), 6.97 (d, *J* = 9.4 Hz, 1H).

^13^C-NMR (126 MHz, Chloroform-*d*) δ 166.65, 163.11, 151.17, 150.61, 149.04, 145.98, 142.30, 141.16, 139.63, 134.89, 134.04, 132.70, 132.46, 13.55, 131.16, 128.94, 128.79, 127.27, 126.42, 126.39, 126.10, 126.08, 22.76, 122.51, 117.80, 113.81, 113.79.

^19^F-NMR (470 MHz, Chloroform-*d*) δ −62.53.

MS (ESI): Exact mass for C_31_H_19_F_3_N_4_O_2_ = 536.14 Found (M + 1) = *m/z* 537.11.

#### 4.1.5. Synthesis of *N*-(5-(2-Oxo-1-(3-(trifluoromethyl)phenyl)-1,2-dihydrobenzo[h][1,6] naphthyridin-9-yl)pyridin-2-yl)methanesulfonamide (Compound **16**)


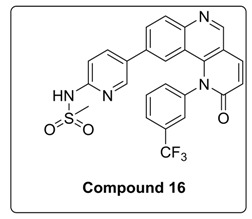


To a solution of compound **11** (0.050 g, 0.1 mmol) in dried DCM (2 mL) at 0 °C was added pyridine (8.6 μL, 1.1 mol) followed by addition of methyl sulphonyl chloride (9 μL, 0.1 mmol) under N_2_ atmosphere. The reaction mixture was heated at 40 °C for 5 h and then cooled to room temperature. The resulting mixture was directly concentrated under high vacuum. To the resulting residue, water was added, and the mixture was extracted twice with ethyl acetate. The combined organic layers extract was washed with brine and dried over anhydrous Na_2_SO_4_. Solvent was evaporated under reduced pressure. The residue was purified by silica gel column chromatography (100–200 mesh, 10% MeOH: DCM) to afford compound **16** as a light yellow solid.

^1^H-NMR (DMSO-*d*_6_, 500 MHz) δ 10.74 (s, 1H), 9.17 (s, 1H), 8.35 (d, 1H, *J* = 9.5 Hz), 8.17 (s, 1H), 8.04–8.01 (d, 2H, *J* = 8.5 Hz), 8.03–8.01 (m, 2H), 7.90–7.87 (m, 1H), 7.79 (d, 1H, *J* = 9.5 Hz), 7.37 (s, 1H), 7.05 (m, 3H).

^1^H-NMR (CDCl_3_, 500 MHz) δ 9.02 (s, 1H), 8.21 (d, 1H, *J* = 8.5 Hz), 8.03 (d, 2H), 7.92 (d, 1H, *J* = 10.0 Hz), 7.82–7.76 (m, 3H), 7.82–7.7 (m, 1H), 7.59 (d, 1H, *J* = 8.0 Hz), 7.31–29 (m, 1H), 7.16 (d, 1H, *J* = 9.0 Hz), 6.99–6.96 (m, 2H), 3.23 (s, 3H).

^13^C-NMR (126 MHz, Chloroform-*d*) δ 163.08, 152.05, 150.77, 149.07, 143.96, 142.266, 139.63, 138.27, 132.59, 131.96, 131.46, 129.70, 128.44, 126.37, 122.84, 122.59, 40.87.

^19^F-NMR (470MHz, DMSO-d6): δ −61.07.

MS (ESI): Exact mass for C_25_H_17_F_3_N_4_O_3_S = 510.09, Found (M + 1) = *m/z* 511.17.

#### 4.1.6. Synthesis of 9-Bromo-1-(3-(trifluoromethyl)phenyl)-1,4-dihydro-2H-[1,3]oxazino[5,4-c]quinolin-2-one and 9-morpholino-1-(3-(trifluoromethyl)phenyl)-1,4-dihydro-2H-[1,3]oxazino[5,4-c]quinolin-2-one (Compound **17**–**18**) 

To the stirred solution of corresponding alcohol in 1,4-dioxane was added diphosgene at 0 °C under N_2_ atmosphere. After that, reaction was allowed to stir at room temperature for 6 h. The progress of the reaction was monitored using thin layer chromatography (solvent: 70% ethyl acetate: Hexane). Following that, ice was added to the reaction mixture, and the mixture was extracted with ethyl acetate. The combined organic layers extract was washed with brine and dried over anhydrous Na_2_SO_4_. Solvent was evaporated under reduced pressure. The resulting mixture was purified by column chromatography (the compound was eluted in 95% ethyl acetate: Hexane) to afford compound **17** and **18** as yellow solid.


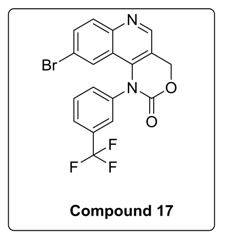


^1^H-NMR (500 MHz, DMSO-*d*_6_) δ 9.07 (s, 1H), 8.09 (d, *J* = 9.1 Hz, 1H), 8.05 (s, 1H), 7.95 (d, *J* = 8.0 Hz, 1H), 7.90 (d, *J* = 8.2 Hz, 2H), 7.84 (d, *J* = 8.1 Hz, 1H), 6.84 (s, 1H), 5.75 (s, 2H).

^13^C-NMR (126 MHz, DMSO-*d*_6_) δ 152.07, 147.00, 140.59, 133.23, 132.97, 131.50, 131.10, 126.12, 125.76, 125.60, 119.95, 119.18, 117.74, 66.01.

^19^F-NMR (470 MHz, DMSO-*d*_6_) δ −61.20.

MS (ESI): Exact mass for C_18_H_10_BrF_3_N_2_O_2_ = 421.99, Found = *m/z* 423.02.


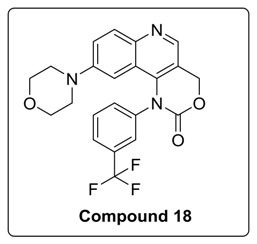


^1^H-NMR (500 MHz, DMSO-*d6*) δ 8.82 (s, 1H), 8.12 (s, 1H), 8.02 (d, *J* = 8.7 Hz, 1H), 7.89 (t, *J* = 8.2 Hz, 2H), 7.76 (s, 3H), 7.11 (s, 1H), 6.94 (s, 1H), 6.45–6.37 (m, 1H), 6.17 (s, 2H), 5.69 (d, *J* = 4.0 Hz, 2H).

^13^C-NMR (126 MHz, DMSO-*d6*) δ 160.00, 152.81, 148.87, 146.60, 146.46, 141.37, 140.87, 135.96, 135.33, 132.64, 131.20, 131.00, 127.74, 122.83, 118.64, 118.33, 117.47, 108.28, 66.25.

^19^F-NMR (470 MHz, DMSO-*d6*) δ −56.33. 

MS (ESI): Exact mass for C_22_H_18_F_3_N_3_O_3_ = 429.13, Found (M + 1) = *m/z* 430.16.

#### 4.1.7. Synthesis of 9-(6-Aminopyridin-3-yl)-1-(3-(trifluoromethyl)phenyl)-1,4-dihydro-2H-[1,3]oxazino[5,4-c]quinolin-2-one (Compound **19**)


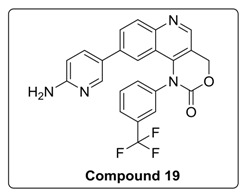


To a solution of compound **17** (0.1 g, 0.25 mmol) in 1,4-dioxane (2 mL) at room temperature in a sealed tube was added corresponding pyridyl boronic ester (0.0498 g, 0.25 mmol), Pd(Ph_3_P)_3_Cl_2_ (0.023 mmol), and K_2_CO_3_ (1N, 0.50 mmol) under N_2_ atmosphere. The reaction mixture was degassed for 5–10 min. After degassing, the reaction mixture was heated at 100 °C for 15 h and then cooled to room temperature. The resulting mixture was directly concentrated under high vacuum. To the resulting residue, ice-cold water was added, and the mixture was extracted twice with ethyl acetate. The combined organic layers extract was washed with brine and dried over anhydrous Na_2_SO_4_. Solvent was evaporated under reduced pressure. The resulting mixture was purified by column chromatography (The compound was eluted at 5% MeOH: DCM) to afford compound **19** as yellow solid.

^1^H-NMR (500 MHz, Chloroform-d) δ 8.51 (s, 1H), 7.90–7.87 (m, 2H), 7.57 (d, *J* = 6.2 Hz, 1H), 7.50–7.49 (m, 2H), 7.30–7.24 (m, 1H), 6.03 (d, *J* = 2.6 Hz, 1H), 5.42 (s, 2H), 3.64 (t, *J* = 4.8 Hz, 4H), 2.67 (t, *J* = 4.8 Hz, 4H).

^13^C-NMR (126 MHz, Chloroform-*d*) δ 148.87, 142.98, 140.94, 131.48, 130.36, 130.37, 130.04, 124.93, 121.71, 116.65, 104.49, 66.36, 66.10, 48.51, 29.70.

^19^F-NMR (470 MHz, Chloroform-*d*) δ −62.64.

MS (ESI): Exact mass for C_23_H_15_F_3_N_4_O_2_ = 436.11, Found (M + 1) = *m/z* 437.11.

### 4.2. In Silco Studies

#### 4.2.1. Homology Modeling of ATR Kinase

SWISS-MODEL [12] workspace was used to build three-dimensional protein structure of ATR kinase using experimentally determined crystal structures of related family members as templates. ATR amino acid sequence was retrieved from NCBI (GenBank ID: CAA70298.1) database in FASTA format. It consists of 2644 amino acids, among which 2293–2567 belong to the kinase domain of ATR. The FASTA sequence (2293–2567 amino acids) was directly taken into Swiss model work space for ATR kinase structure prediction. The target sequence was searched with BLAST and HHBlits [15] for evolutionary-related protein structures matching the target sequence. Model generation of ATR kinase was carried out based on the target-sequence alignment using ProMod3 version 1.1.0 [16] Model was analyzed for its quality using QMEAN score. QMEAN scores were very low, indicating that the generated model was of good quality and could be used for further studies. Visualization and analysis of the model were done using PyMOL (The PyMOL Molecular Graphics System, Version 1.74 Schrodinger, LLC) and Maestro programmes (Maestro, Schrödinger, LLC) [17].

#### 4.2.2. Model Validation

The quality of ATR kinase homology model was assessed utilizing different online validation tools such as PROCHEK [18], ProSA [19], Superpose 1.0 [13] and Rampage [20]. The PROCHECK online software was utilized to check the stereo chemical quality of the protein by analyzing residue by residue bond length, bond angles, torsional angles, and chirality. ProSA online server was run to check the sequence to structure and validation of predicted 3-D structure based on z-score. Ramachandran plot analysis was performed to visualize energetically allowed regions for backbone ϕ/ψ torsion angles.

#### 4.2.3. MD Simulations

MD simulations were performed using the GROMACS 5.1.2 Package [21] under Amber 99SB-ILDN force field, which added 10 Å TI3P water with periodic boundaries and counter Cl^−^. Equilibration of the system was conducted in two phases, first phase was NVT ensemble in which temperature was maintained at 300 k for 100 ps, and the second phase was NPT ensemble in which pressure was maintained to a reference pressure of 1 bar with a coupling time of 100 ps. The simulations lasted for 20 ns and were carried out at temperature of 300 K. The root mean square distance (RMSD) values were plotted versus the simulation time to judge whether the simulations had reached a level of equilibrium. From the MD simulations, we obtained the average structure of ATR kinase and used it for further docking studies in Maestro V11.2 (Maestro, Schrödinger, LLC).

#### 4.2.4. Docking Studies

In silico docking studies were conducted using the Glide module (XP) of Schrödinger Maestro v11.2 software (Maestro, Schrödinger, LLC) [17]. Docking consists of four steps: Protein Preparation [22], Receptor Grid Generation, Ligand Preparation [22], and Ligand Docking. For each docked ligand, the best docked pose with lowest Glide score value was recorded and compared.

Protein preparation and receptor Grid generation: The putative ATR kinase was minimized using OPLS-2005 force field using standard parameters. After minimization, a 10 Å grid was generated at the predicted active site by selecting specific residues using receptor grid generation tool of Glide utilizing standard parameters of Glide.

### 4.3. Cell Based Studies

#### Materials and Methods

Colon cancer cell line, HCT-116, was received as a kind gift from Dr. Virupakshi Soppina (IIT Gandhinagar). DMEM, fetal bovine serum, pen-strep, trypsin-EDTA and cell extraction buffer were purchased from Invitrogen Corporation (Carlsbad, CA, USA). Complete EDTA-Free protease inhibitor tablets were purchased from Roche (Basel, Switzerland). Sodium dodecyl sulfate, tetramethylenediamine (TEMED), ammonium per sulphate, Tween-20, β-mercatopethanol, bromophenol blue, non-fat milk, and bovine serum albumin were purchased from Sigma-Aldrich (Darmstadt, Germany). Clarity ECL western blotting substrate and immunoblot PVDF western blotting membrane were purchased from Bio-Rad laboratories (Hercules, CA, USA). Rabbit anti-human Chk1 phospho Ser 317 (Cat. 12302), mouse anti-human phospho p70 S6 kinase Thr389 (Cat. 9206) were purchased from Cell Signaling Technology (Danvers, MA, USA), and mouse anti-human β-actin (Cat. SC47778) was purchased from Santa Cruz Biotechnology (Dallas, Texas, USA). Anti-rabbit IgG HRP-linked secondary antibody (Cat. 7074) and anti-mouse IgG HRP-linked secondary antibody (Cat. 7076) were obtained from Cell Signaling Technology (Danvers, MA, USA). CellTiter-Glo^®^ Luminiscent cell viability assay kit was purchased from Promega Corporation (Madison, WI, USA). 96- and 6-well plates were purchased from Corning (New York, NY, USA).

## 5. Conclusions

In summary, we report the design, synthesis, and docking studies of several Torin2 analogs as potential mTOR/ATR inhibitors. We are also reporting our preliminary studies on cell lines to show the efficacy of these molecules. These preliminary studies would immensely help to develop novel molecules that are selective towards ATR kinase in future. In a nutshell, we believe that these inhibitors would have the potential to be explored as a part of combination therapy for sensitization of cancer cells.

## Figures and Tables

**Table 1:** Glide docking score and important residues interacting with inhibitors.

S.no	List of Compounds	Glide XP Score (kcal/mol)	Key Interacting Residues	Hydrogen Bonding Residues	Hydrogen Bonding Distance (Å)
1	Compound **11** (Torin2)	−8.0	TRP 87(π-π stacking), LYS 16, ASN 89	LYS 16, ASN 89	2.00–2.15
2	Compound **12**	−7.2	TRP 87(π-π stacking), LYS 16	LYS 16	2.13
3	Compound **13**	−8.1	TRP 87(π-π stacking), LYS 16, ASN 89, VAL 88, ILE 189, LEU 190	LYS 16, ASN 89, VAL88, ILE 189, LEU 190	2.10–2.95
4	Compound **14**	−7.0	TRP 87(π-π stacking), LYS 16, ASN 89	LYS 16, ASN 89	2.31–2.87
5	Compound **15**	−6.5	TRP 87(π-π stacking), LYS 16, ASP 202	LYS 16, ASP 202	2.07–1.80
6	Compound **16**	−8.2	TRP 87(π-π stacking), LYS 16, ASN 89	LYS 16, ASN 89	2.05–2.25
7	Compound **17**	−3.4	TRP 87(π-π stacking), LYS 16	LYS 16	2.27
8	Compound **18**	−4.0	TRP 87(π-π stacking), LYS 16	LYS 16	2.24
9	Compound **19**	−4.3	TRP 87(π-π stacking), LYS 16, ASN 89	LYS 16, ASN 89	2.35–2.26

## Supplementary Materials

The following are available online. Figure S1–S41.
