# Supplementary material for: Design, Synthesis, and Docking Studies of New Torin2 Analogs as Potential ATR/mTOR Kinase Inhibitors
Source: Molecules. 2018 Apr 24;23(5):992. doi: 10.3390/molecules23050992 (PMC6102578; doi:10.3390/molecules23050992)
Supplement: Supplementary file 1 [file molecules-23-00992-s001.pdf]

Supplementary files

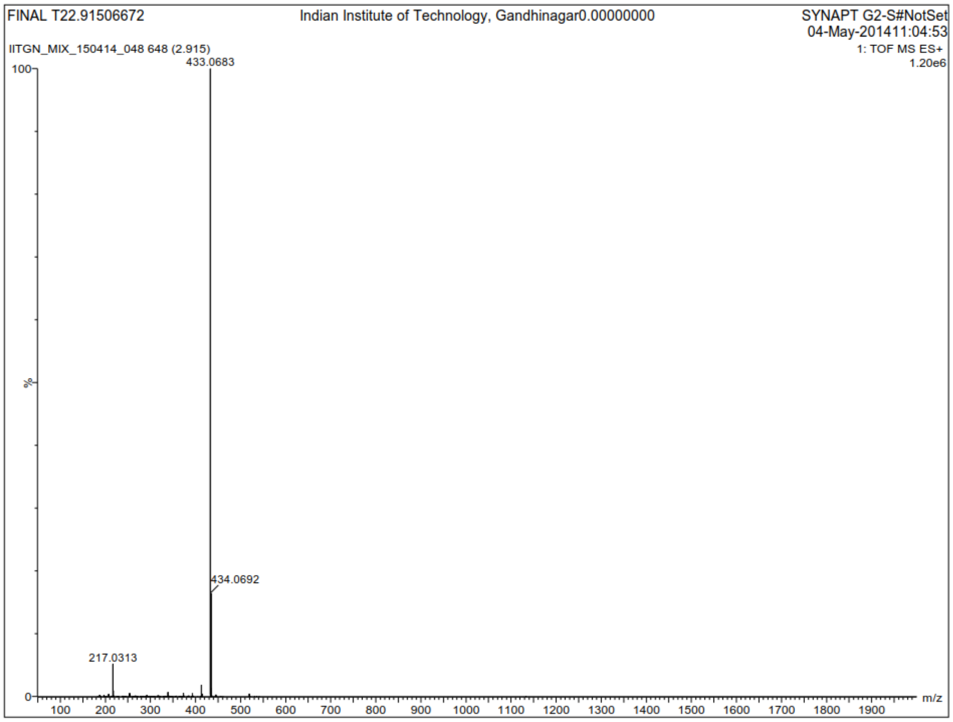

Figure S1: LC-Mass of compound 11

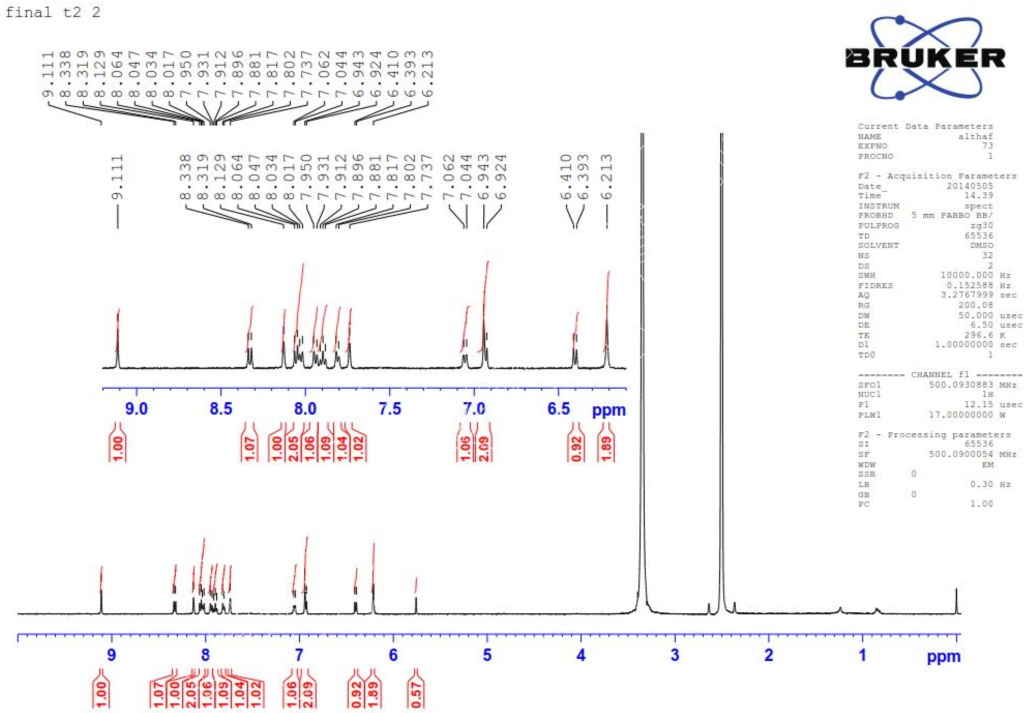

Figure S2: ^{1}H NMR spectra of compound 11

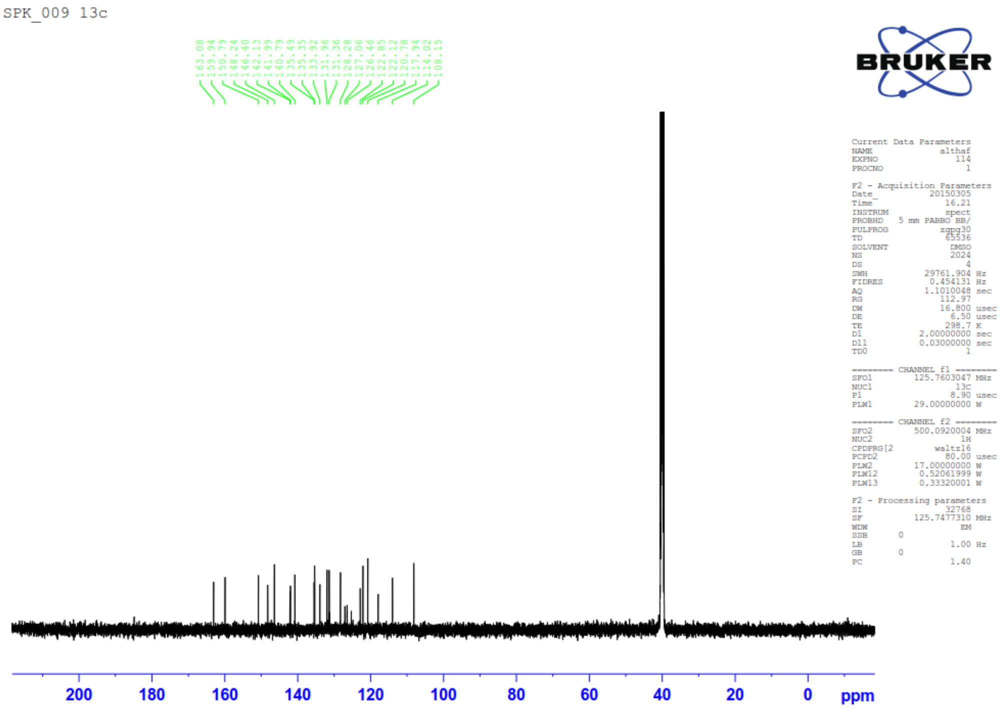

Figure S3: ^{13}C NMR spectra of compound 11

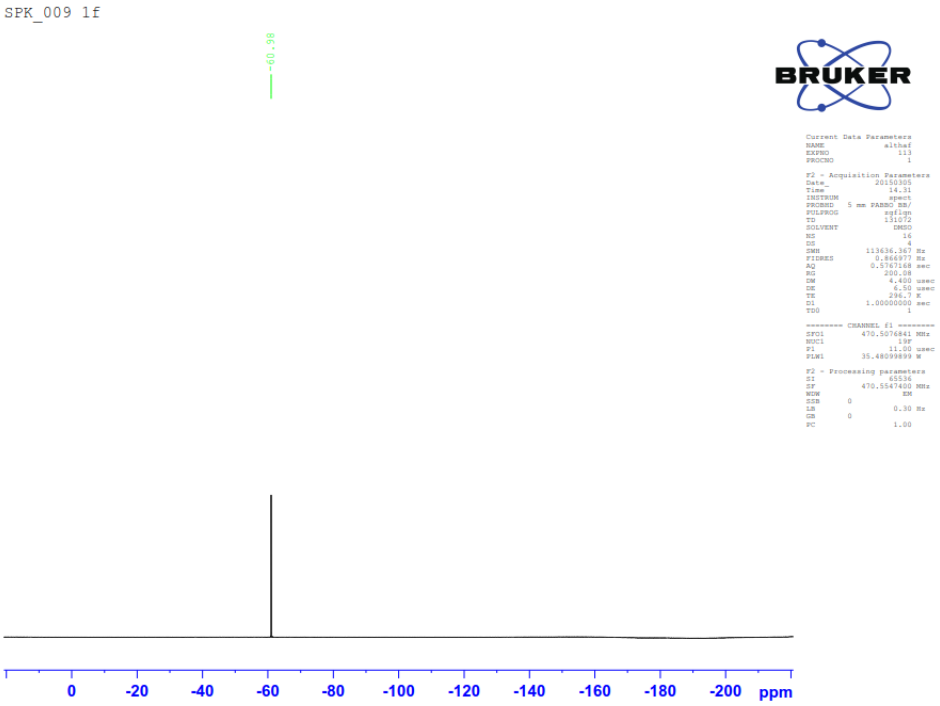

Figure S4: ^{19}F NMR spectra of compound 11

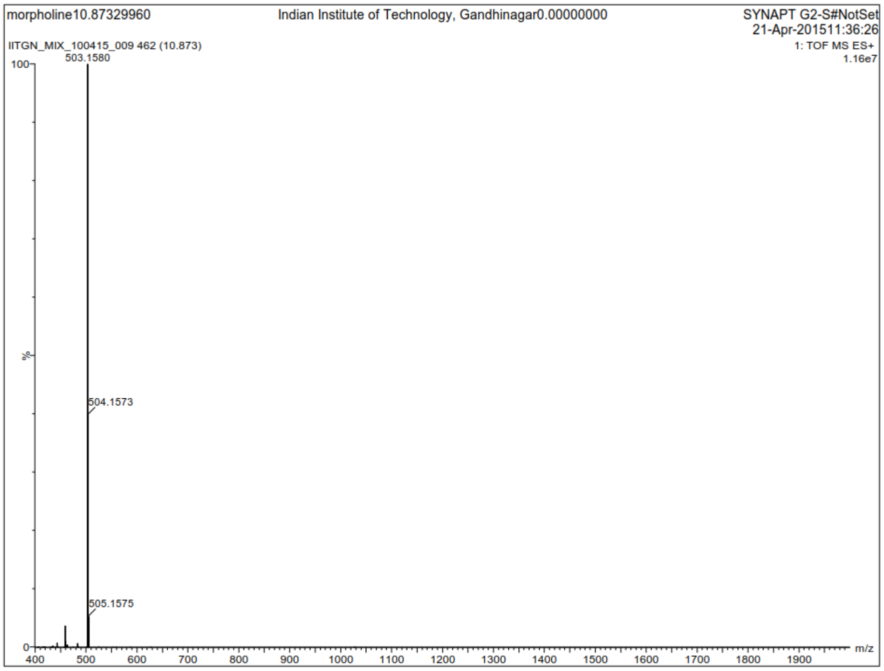

Figure S5: LC-Mass of compound 12

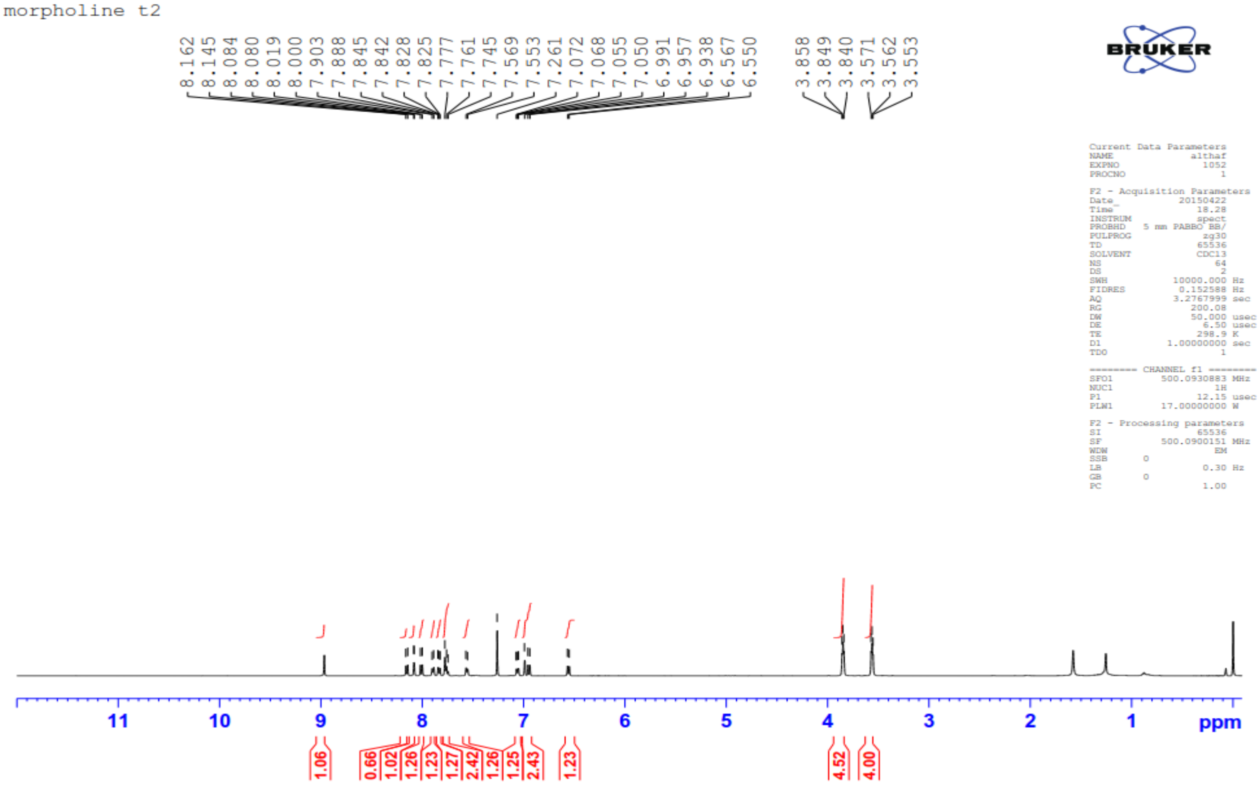

Figure S6: ^{1}H NMR spectra of compound 12

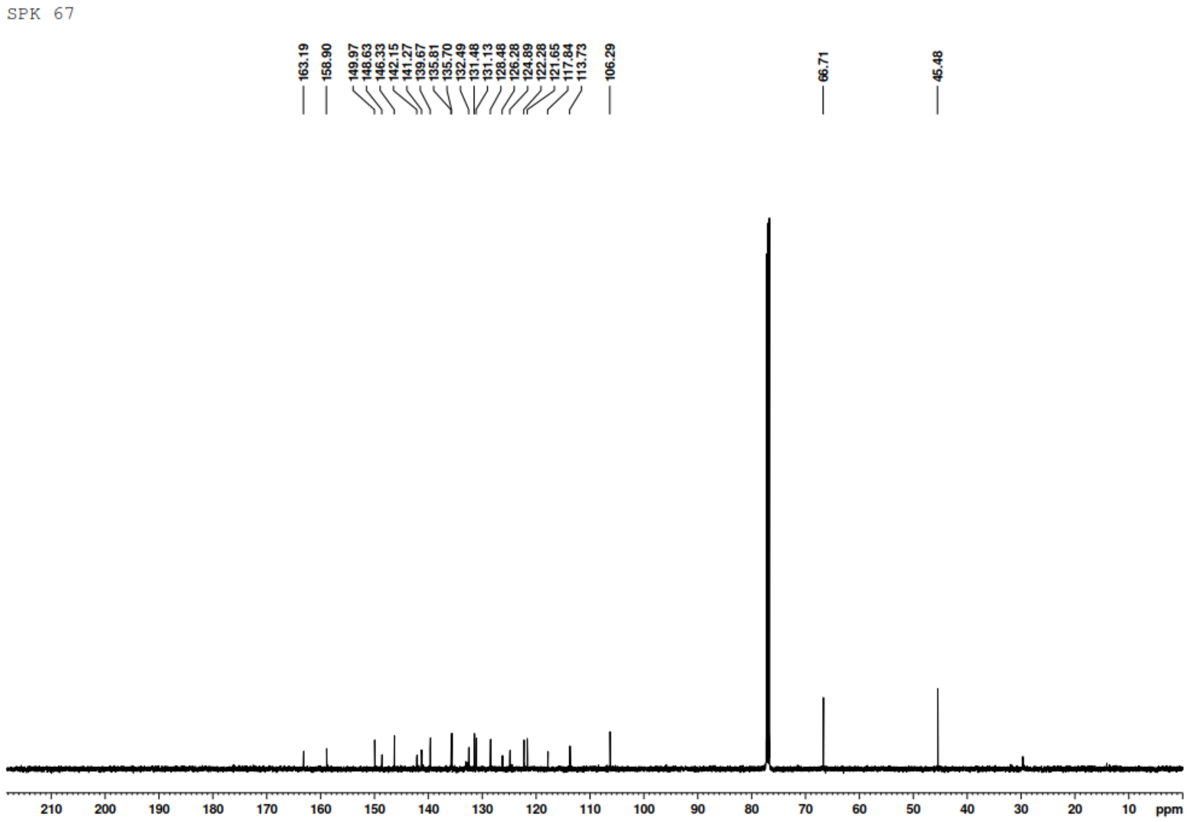

Figure S7: ^{13}C NMR spectra of compound 12

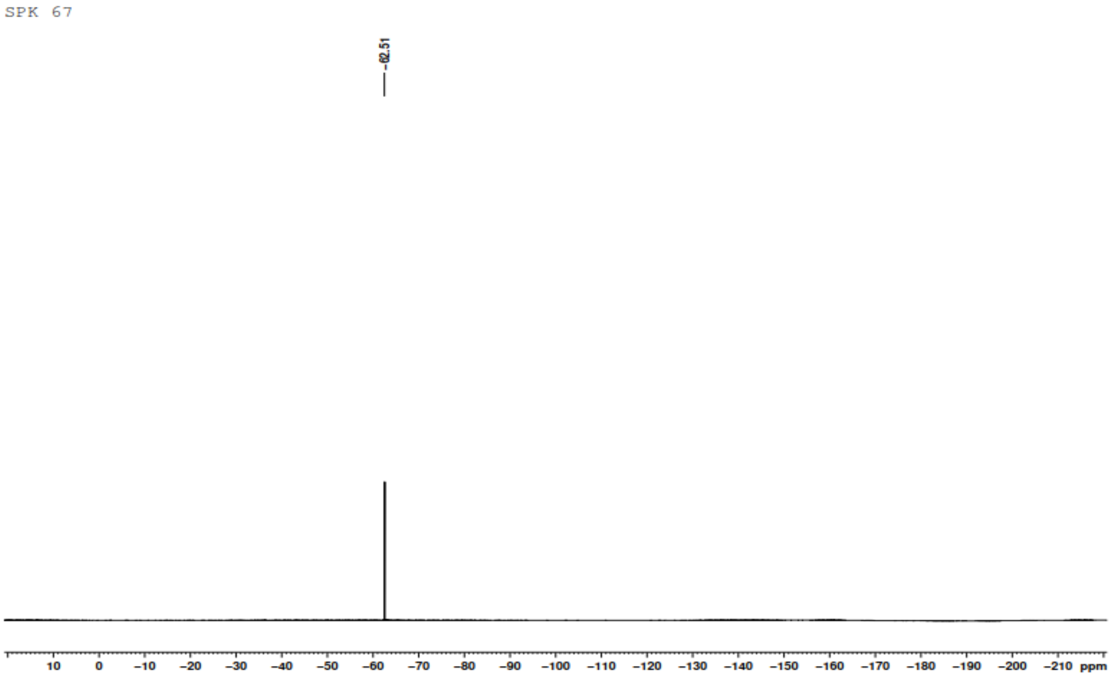

Figure S8: ^{19}F NMR spectra of compound 12

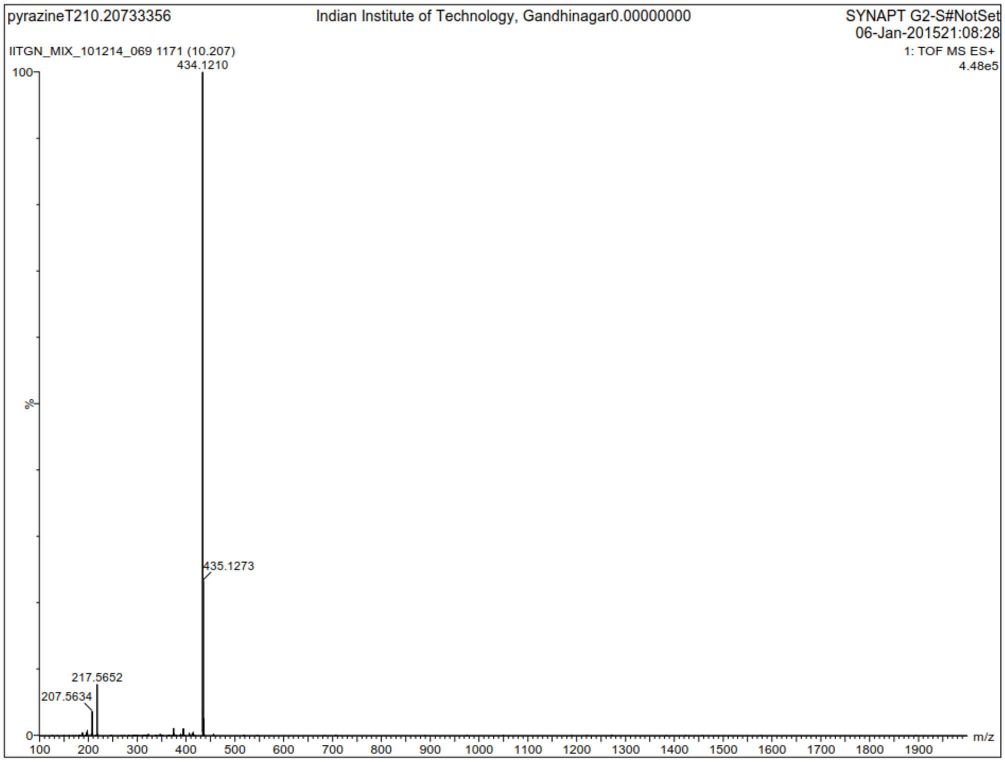

Figure S9: LC-Mass of compound 13

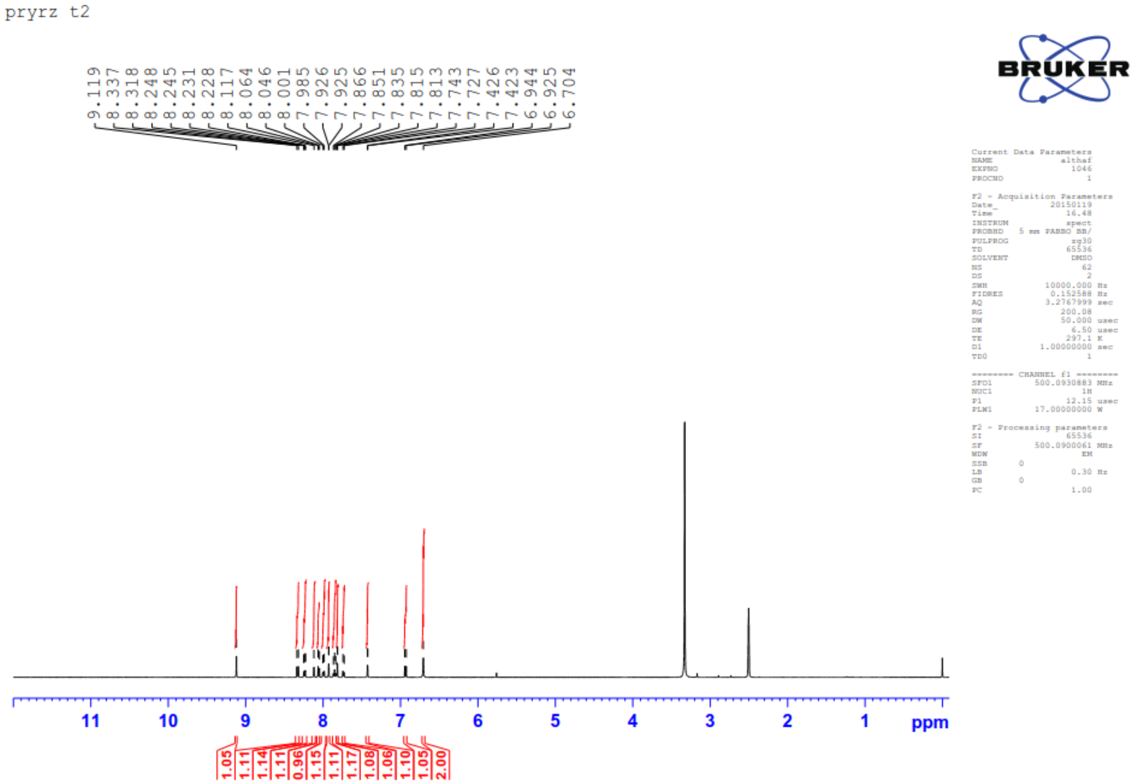

Figure S10: ^{1}H NMR spectra of compound 13

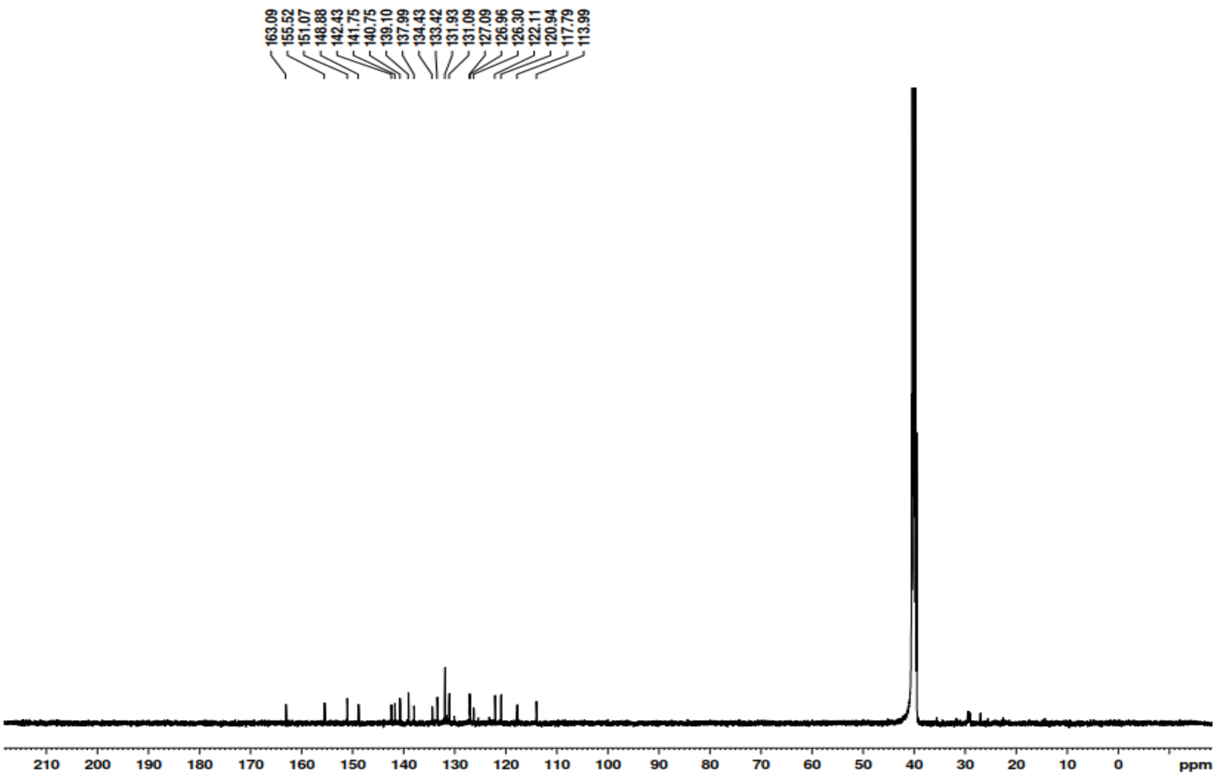

Figure S11: ^{13}C NMR spectra of compound 13

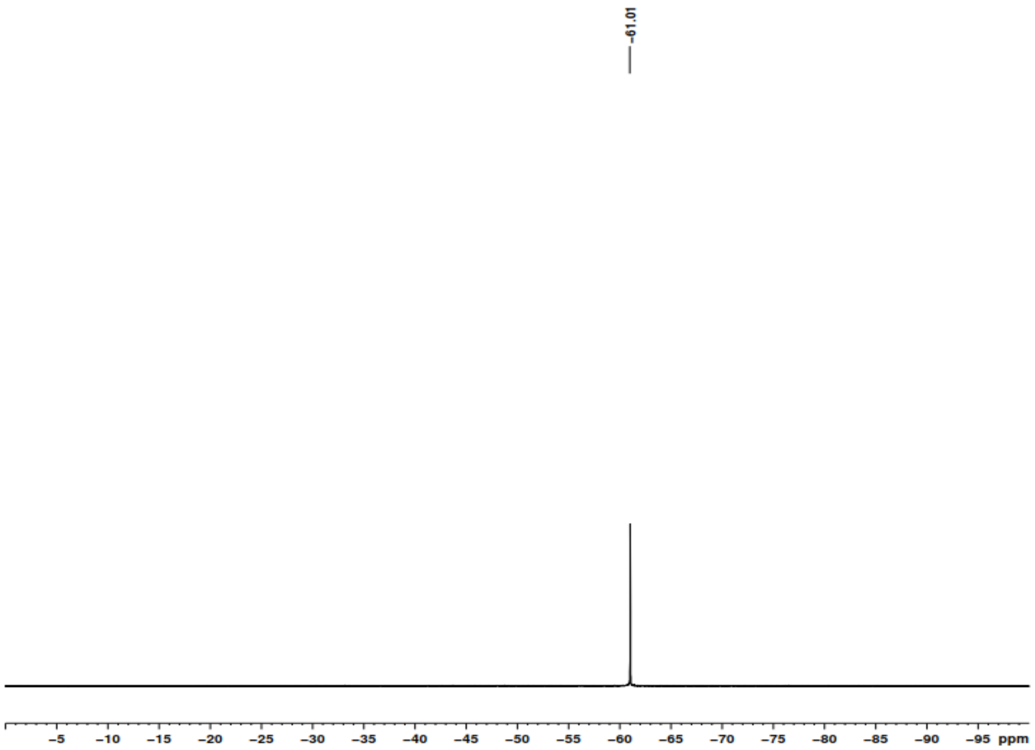

Figure S12: ^{19}F NMR spectra of compound 13

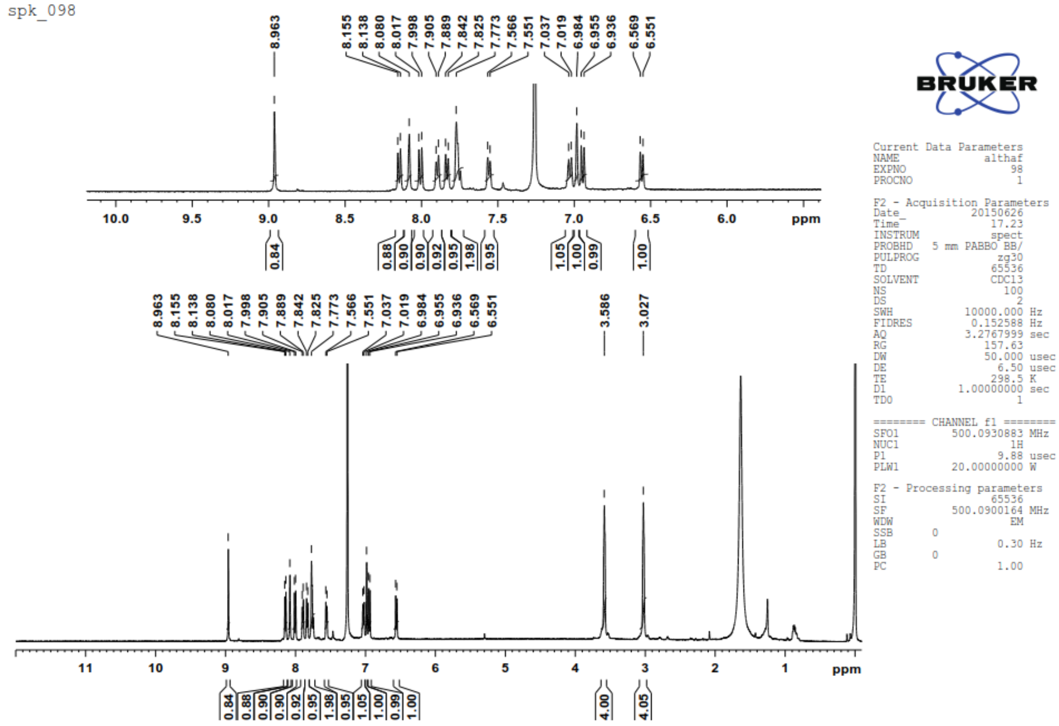

Figure S13: ^{1}H NMR spectra of compound 14

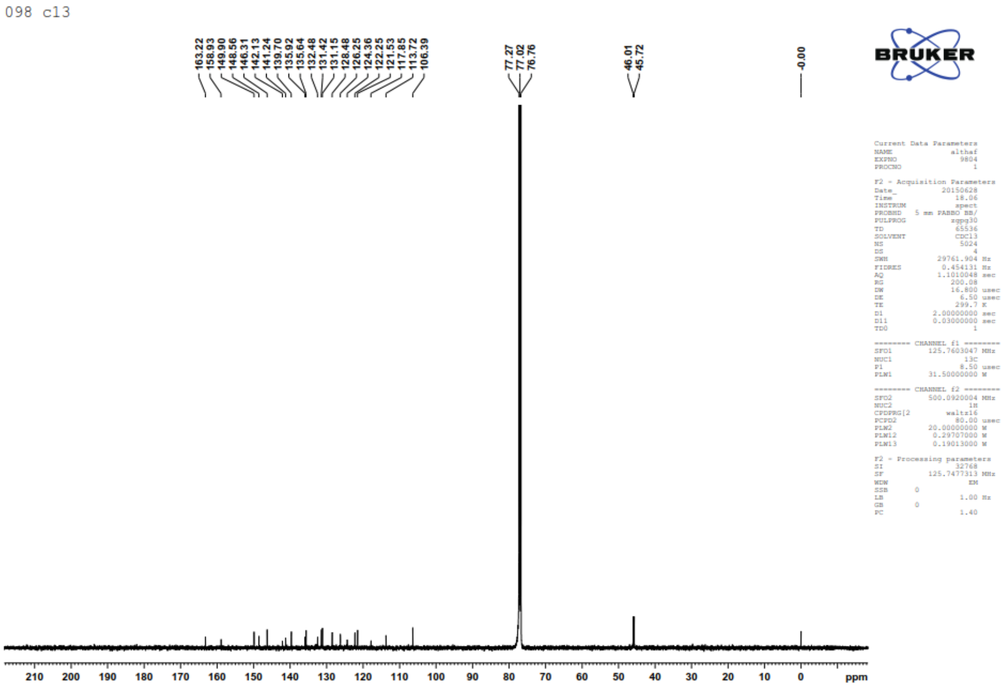

Figure S14: ^{13}C NMR spectra of compound 14

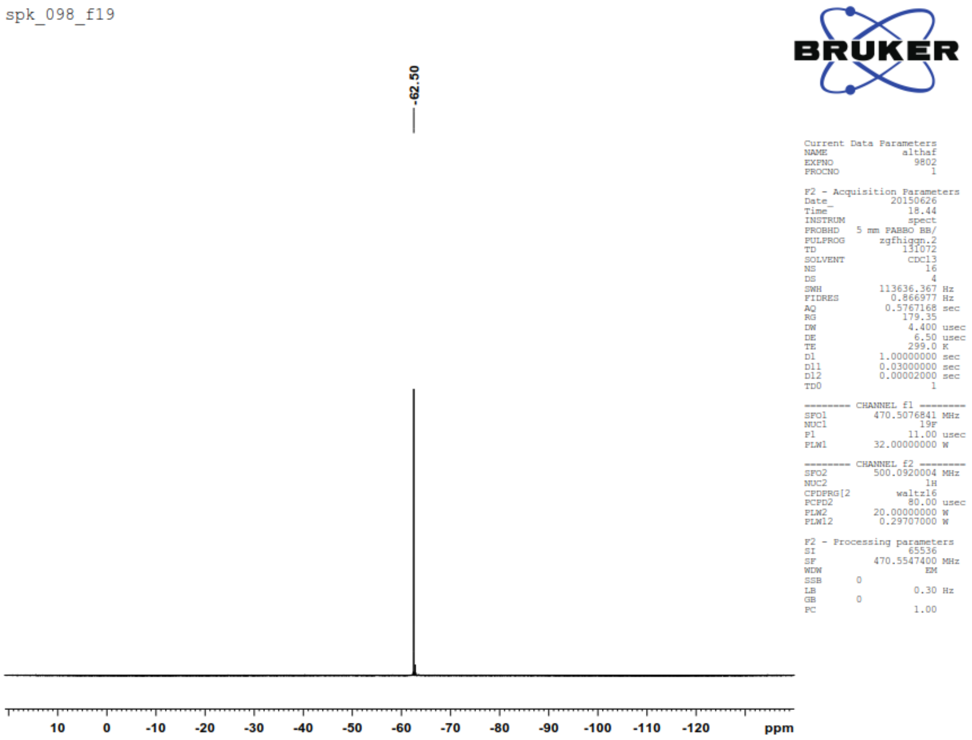

Figure S15: ^{19}F NMR spectra of compound 14

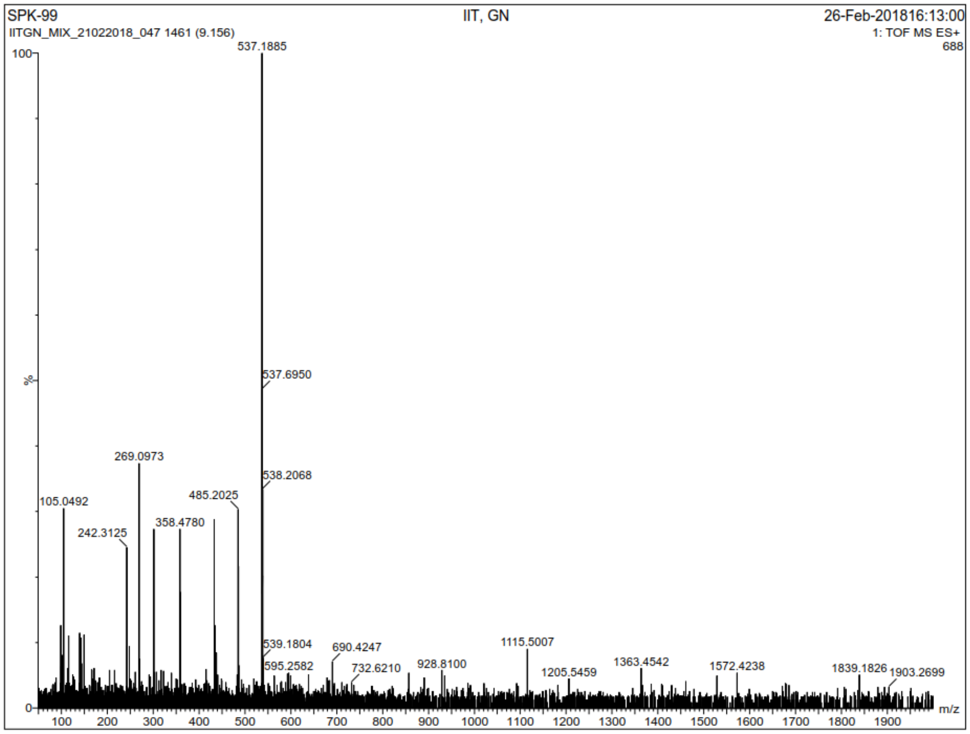

Figure S16: LC-Mass of compound 15

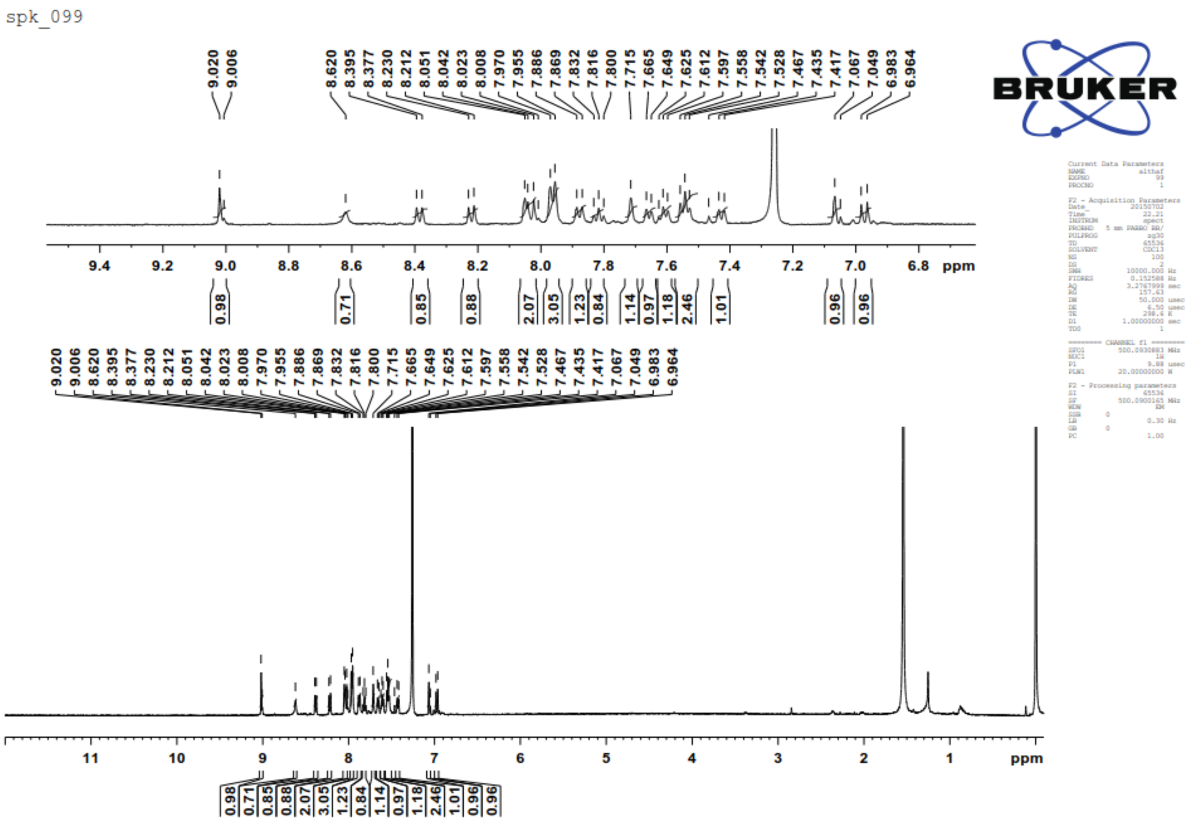

Figure S17: ^{1}H NMR spectra of compound 15

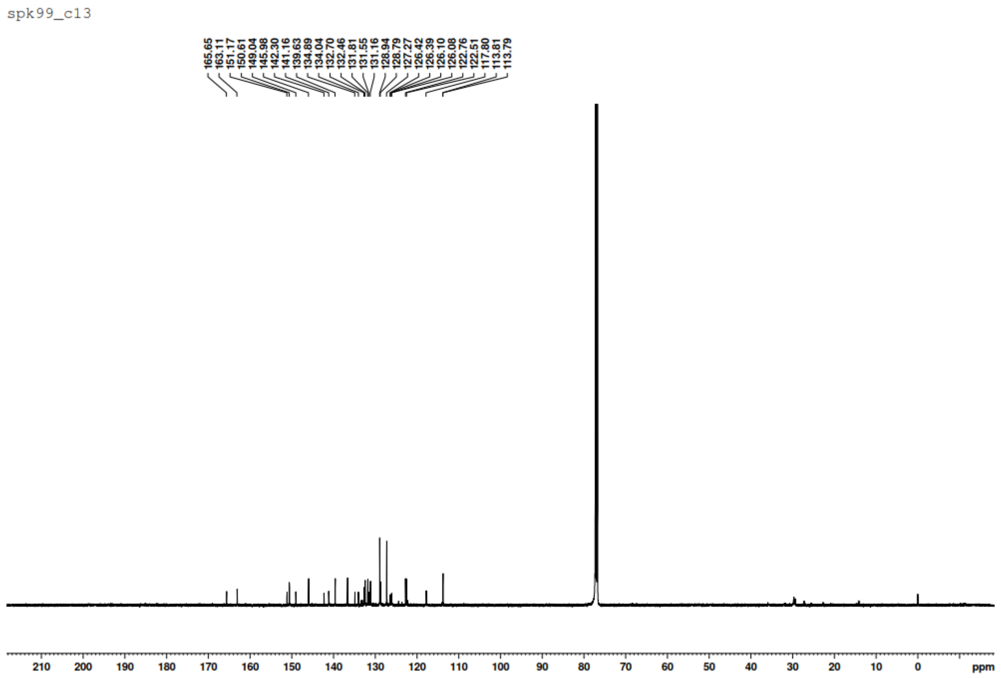

Figure S18: ^{13}C NMR spectra of compound 15

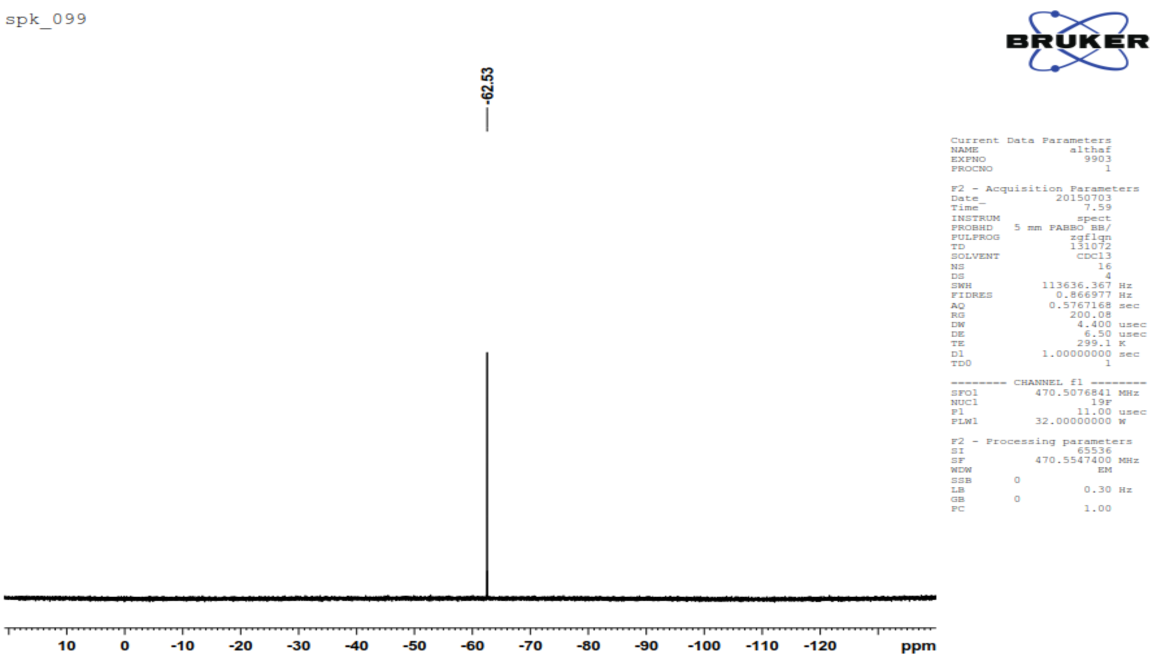

Figure S19: ^{19}F NMR spectra of compound 15

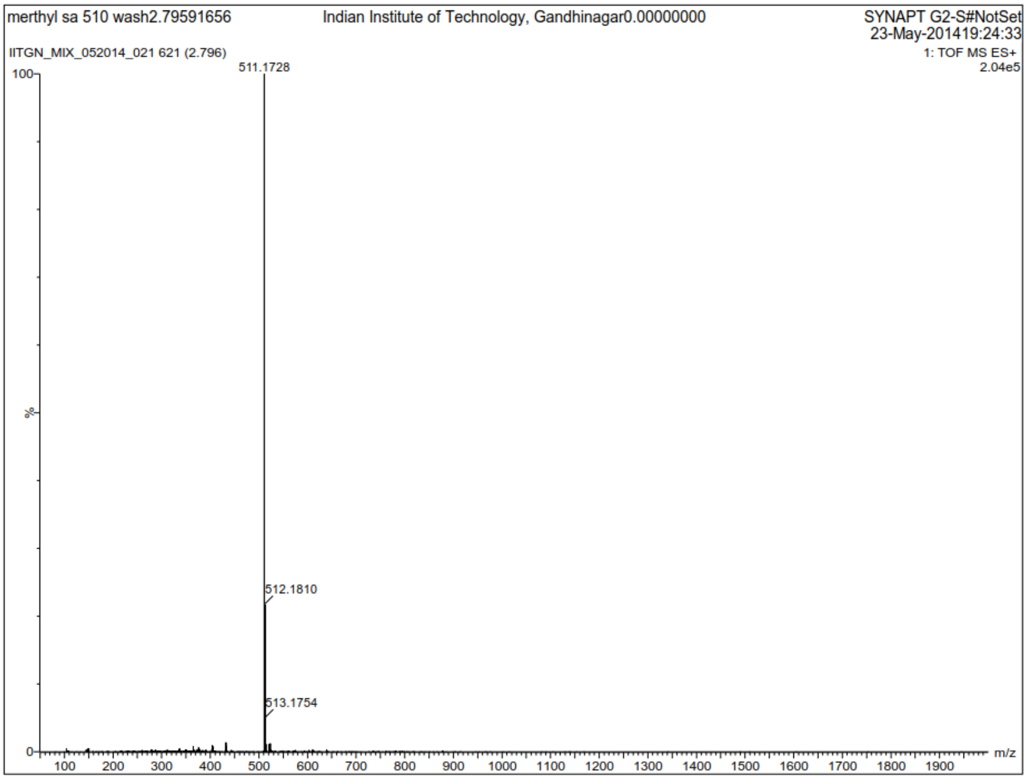

Figure S20: LC-Mass of compound 16

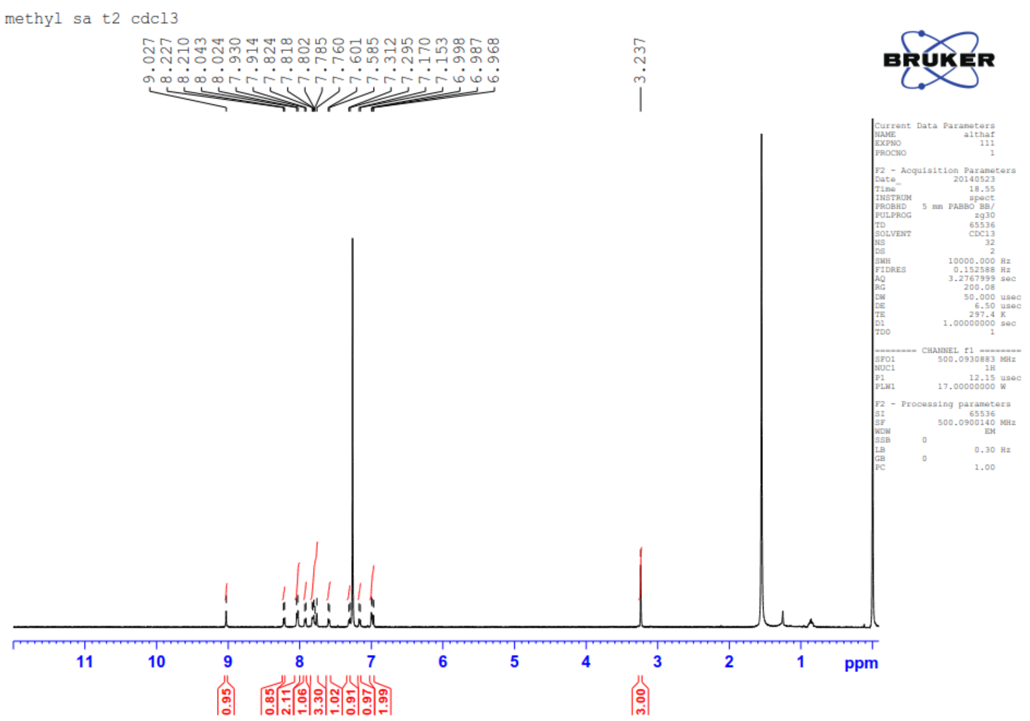

Figure S21: ¹H NMR spectra of compound 16 ($CDCl_3$)

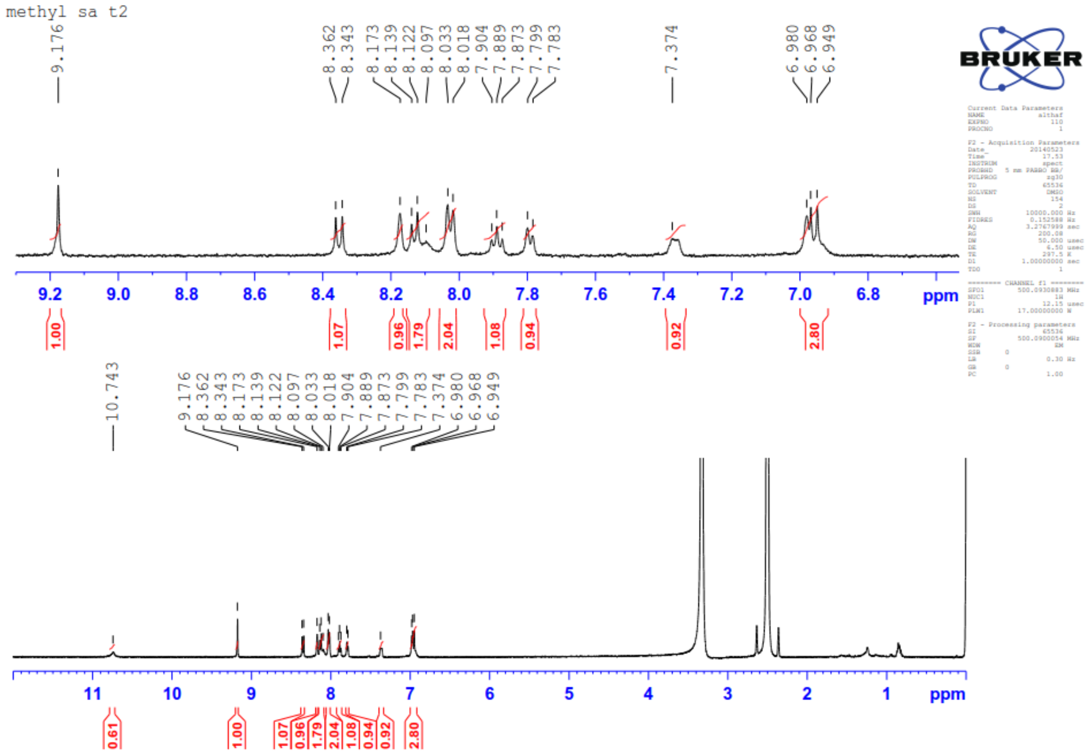

Figure S22: ¹H NMR spectra of compound 16 (DMSO)

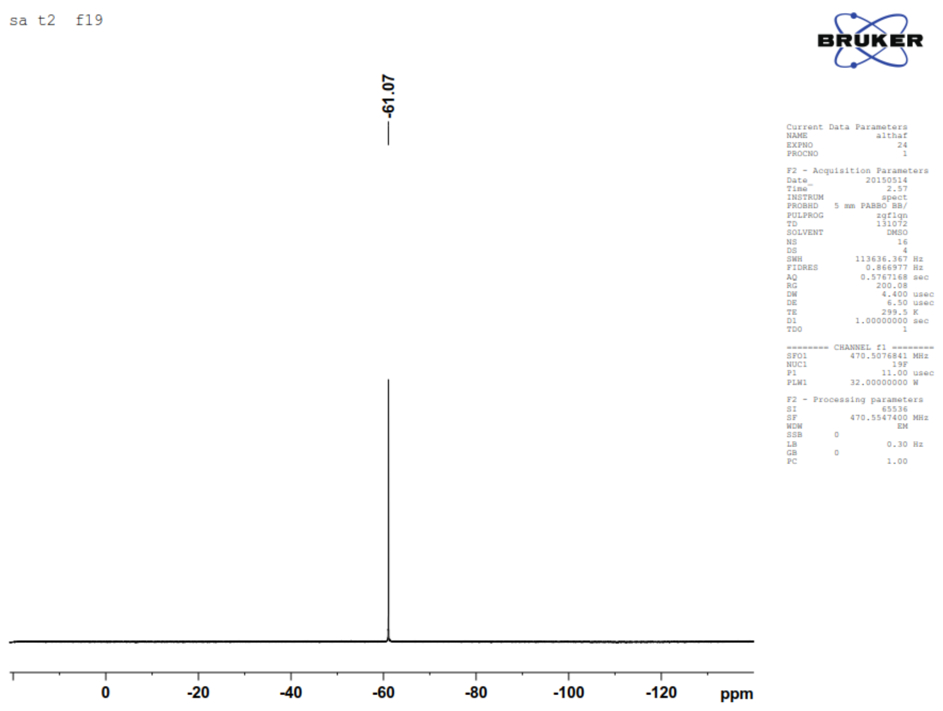

Figure S23: ^{19}F NMR spectra of compound 16 (DMSO-d6)

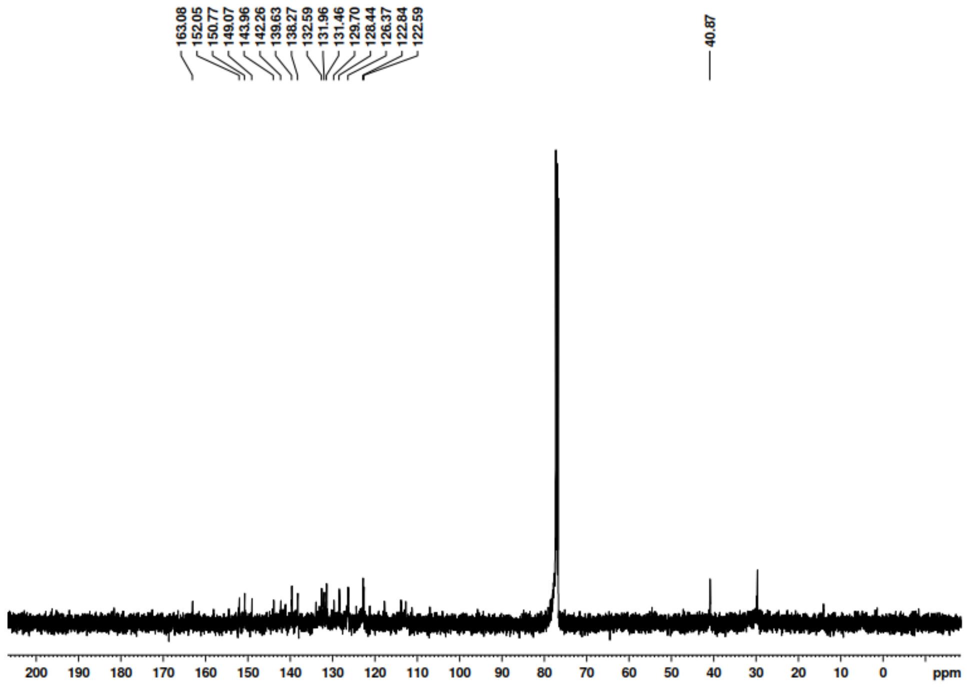

Figure S24: ^{13}C NMR spectra of compound 16 ($CDCl_3$)

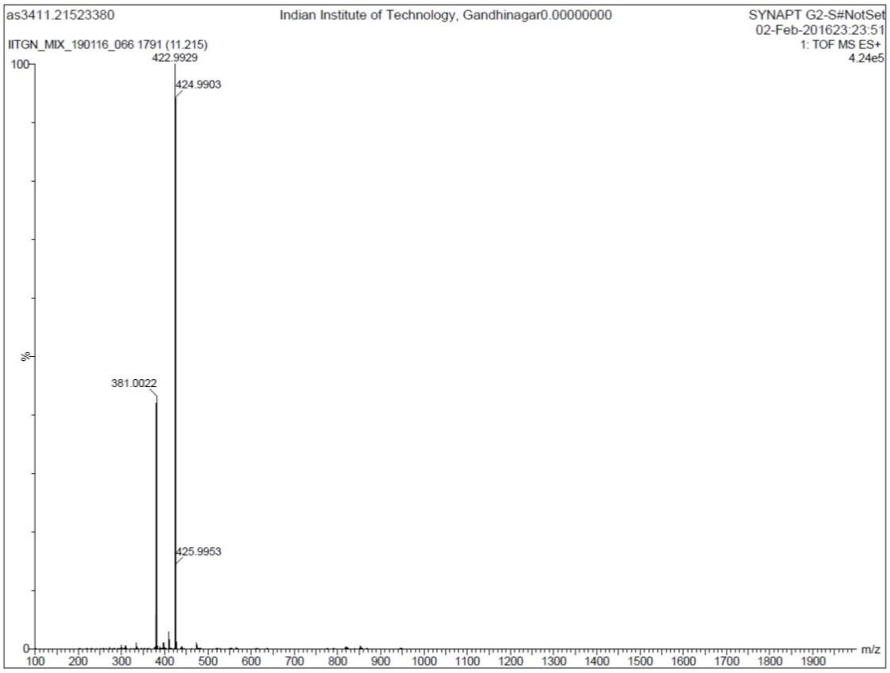

Figure S25: LC-Mass of compound 17

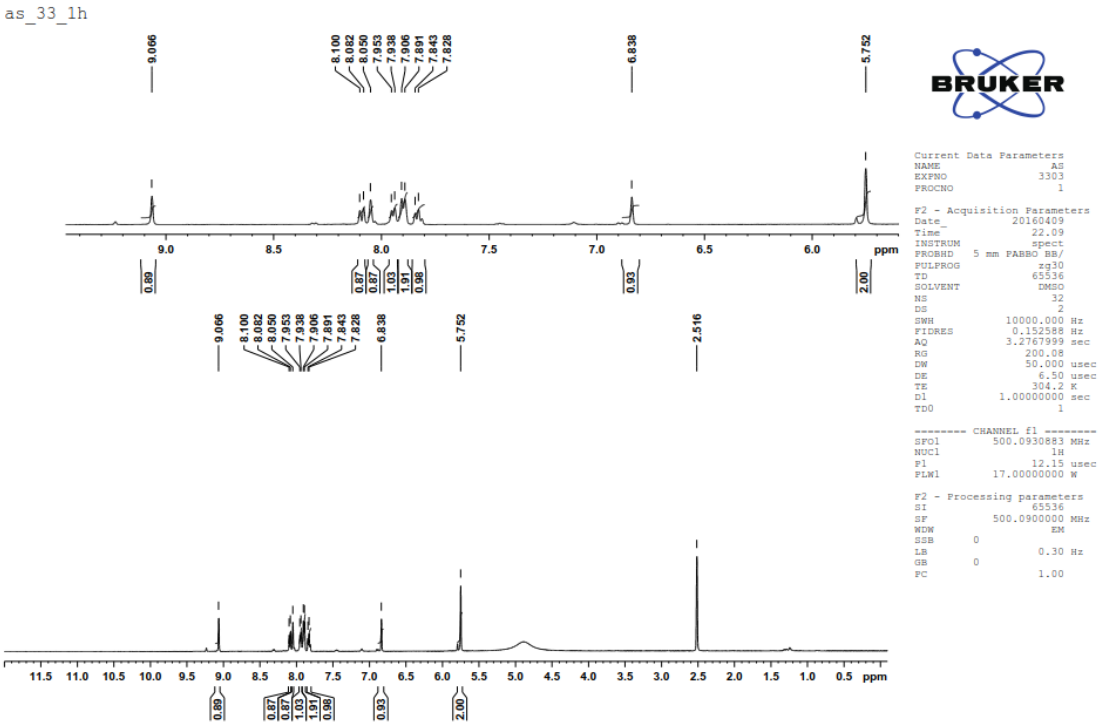

Figure S26: ^{1}H NMR spectra of compound 17

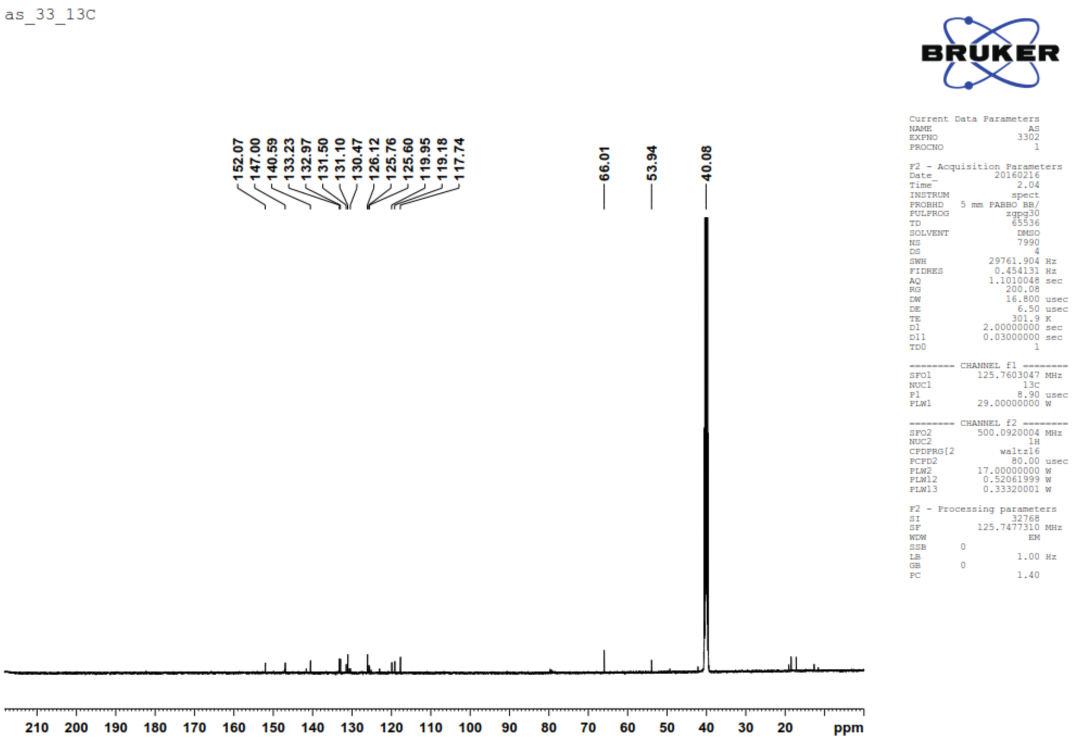

Figure S27: ^{13}C NMR spectra of compound 17

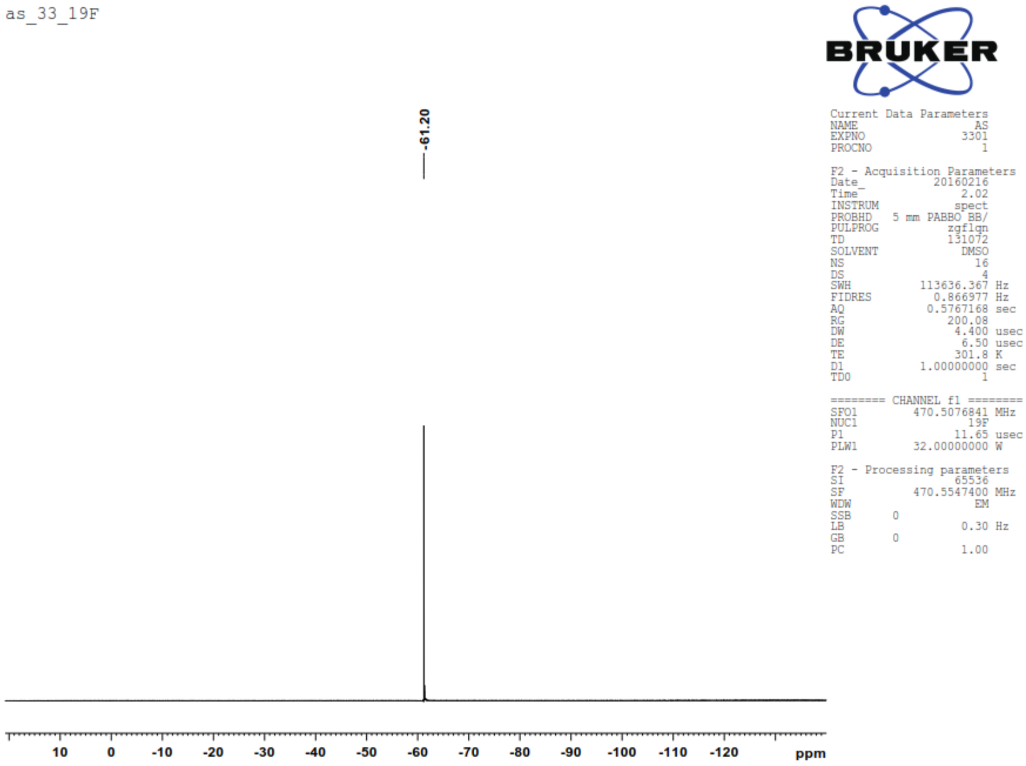

Figure S28: ^{19}F NMR spectra of compound 17

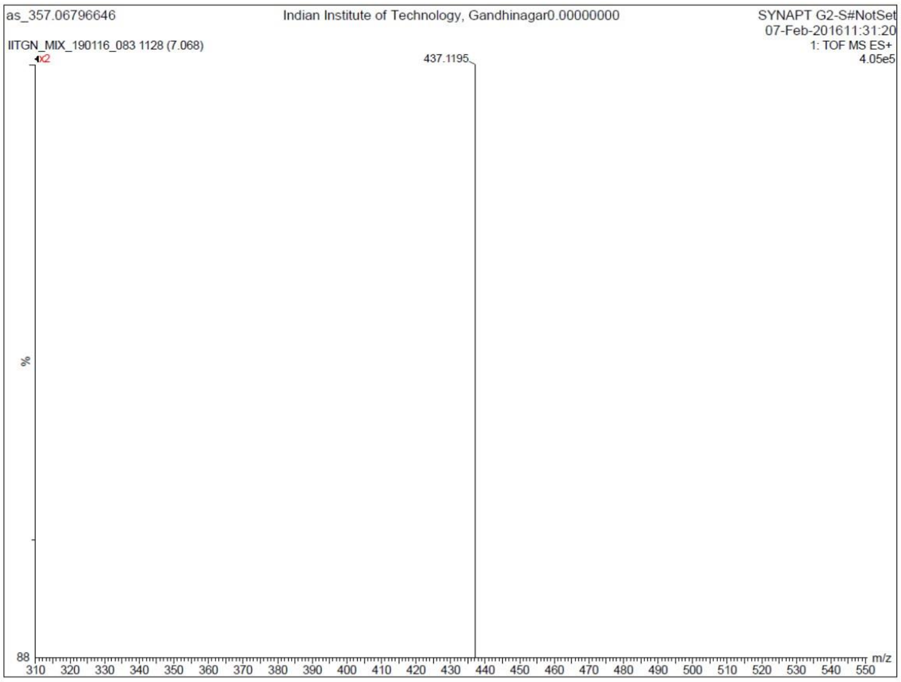

Figure S29: LC-Mass of compound 18

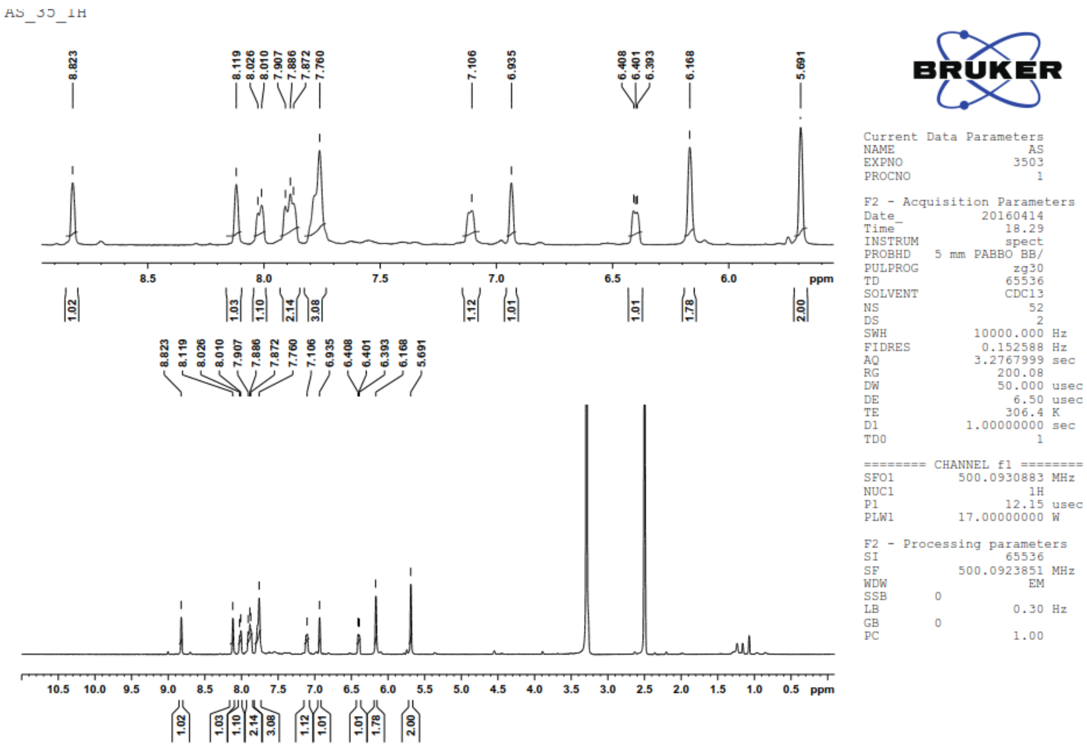

Figure S30: ^{1}H NMR spectra of compound 18

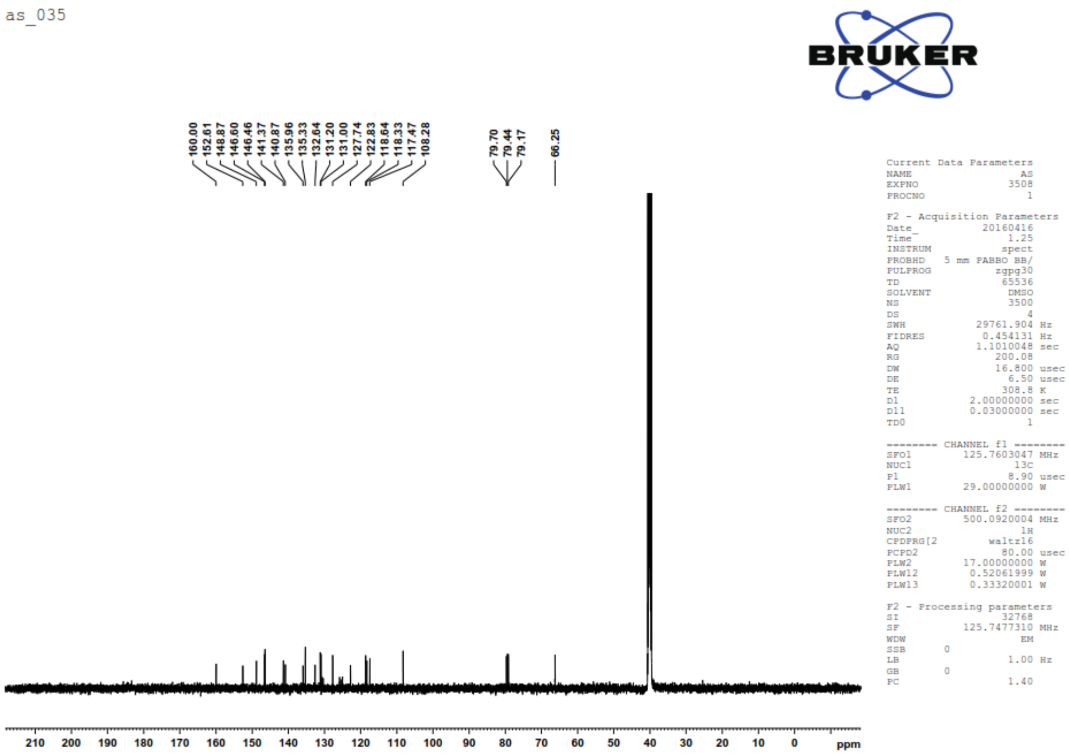

Figure S31: ^{13}C NMR spectra of compound 18

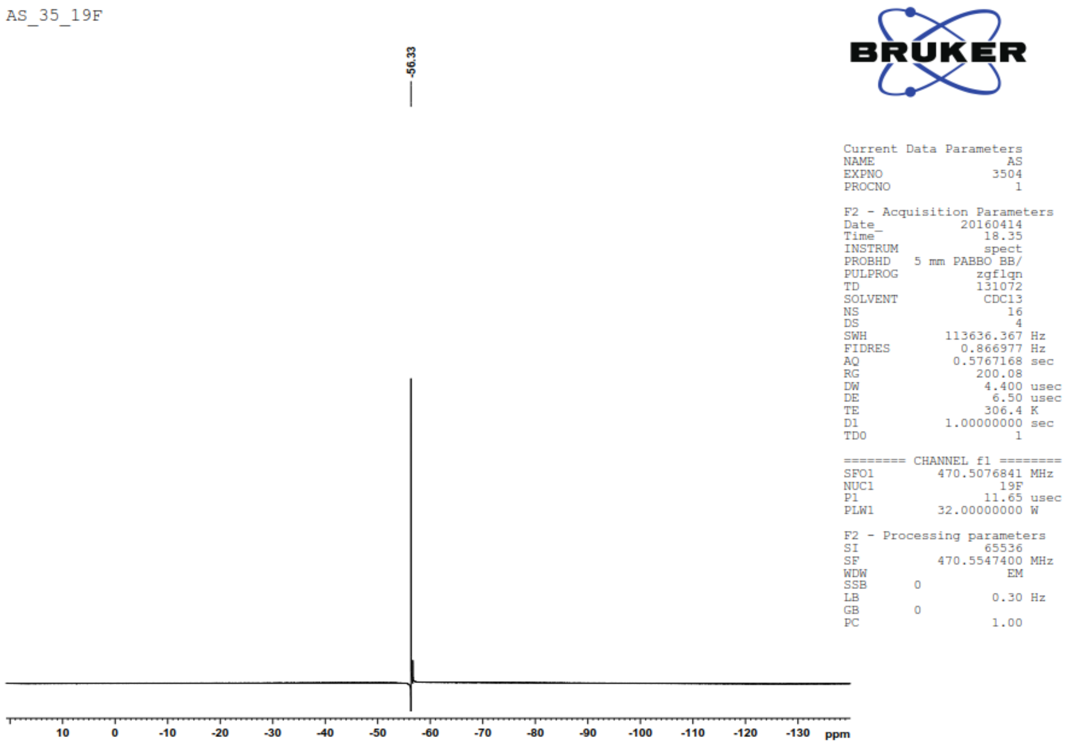

Figure S32: ^{19}F NMR spectra of compound 18

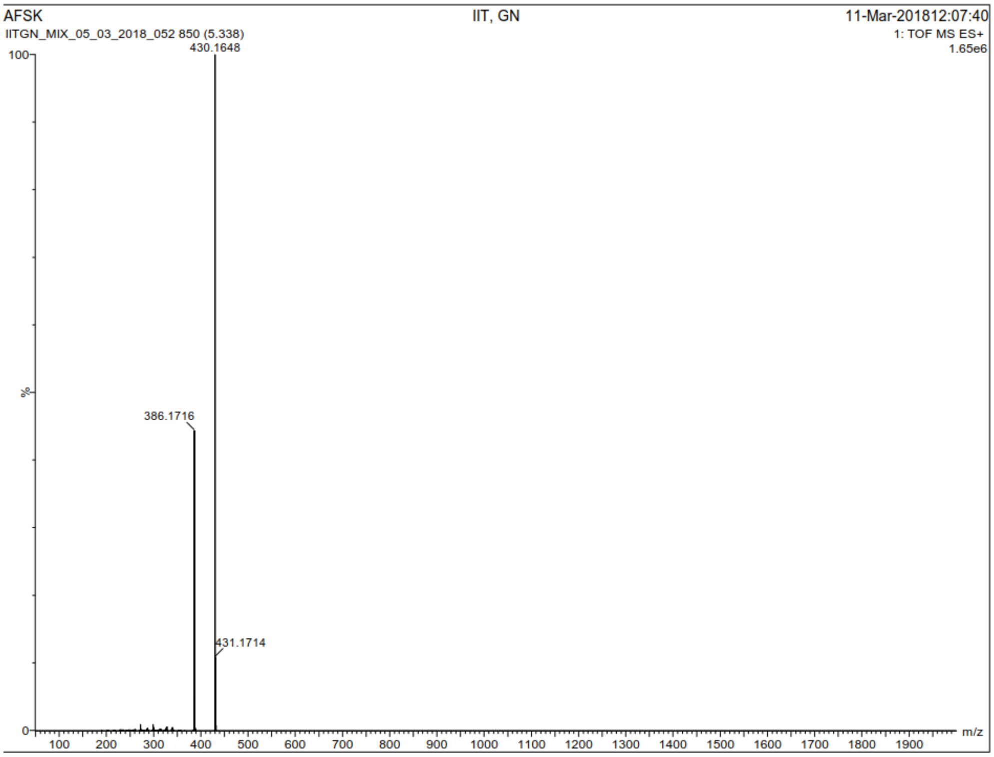

Figure S33: LC-Mass of compound 19

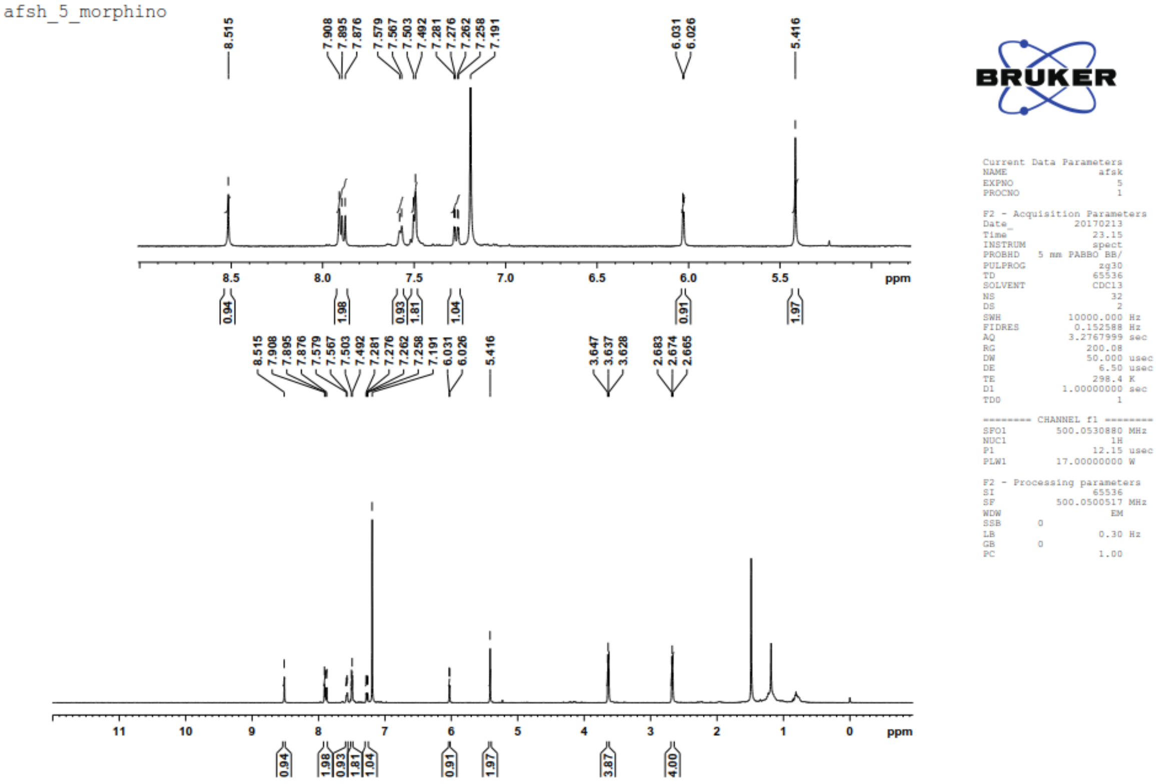

Figure S34: ¹H NMR spectra of compound 19

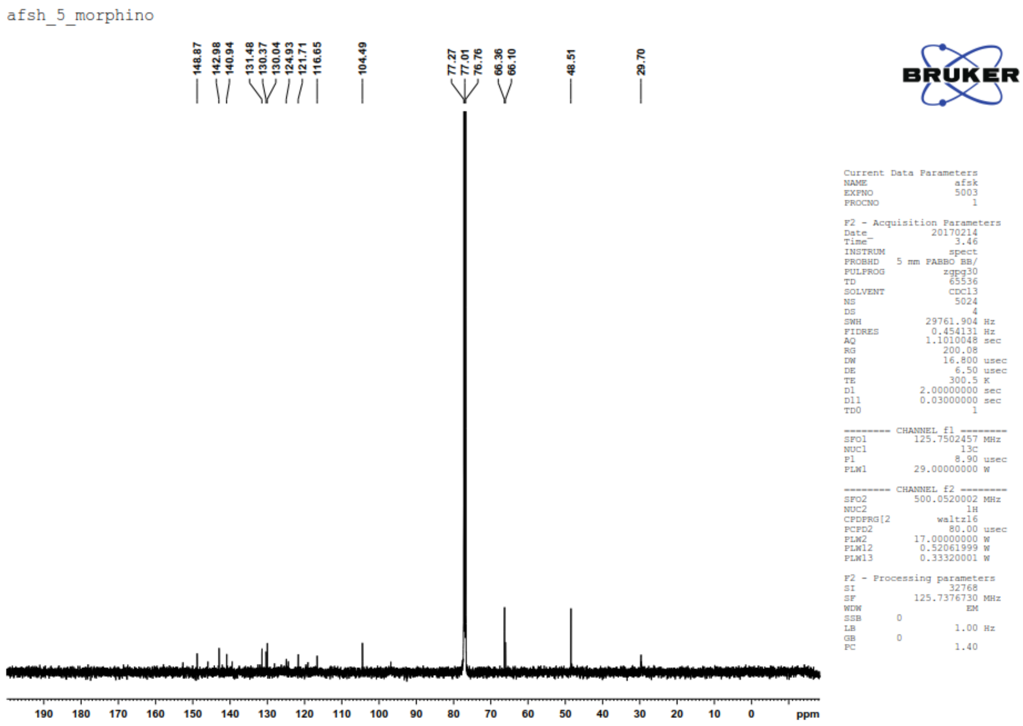

Figure S35: ^{13}C NMR spectra of compound 19

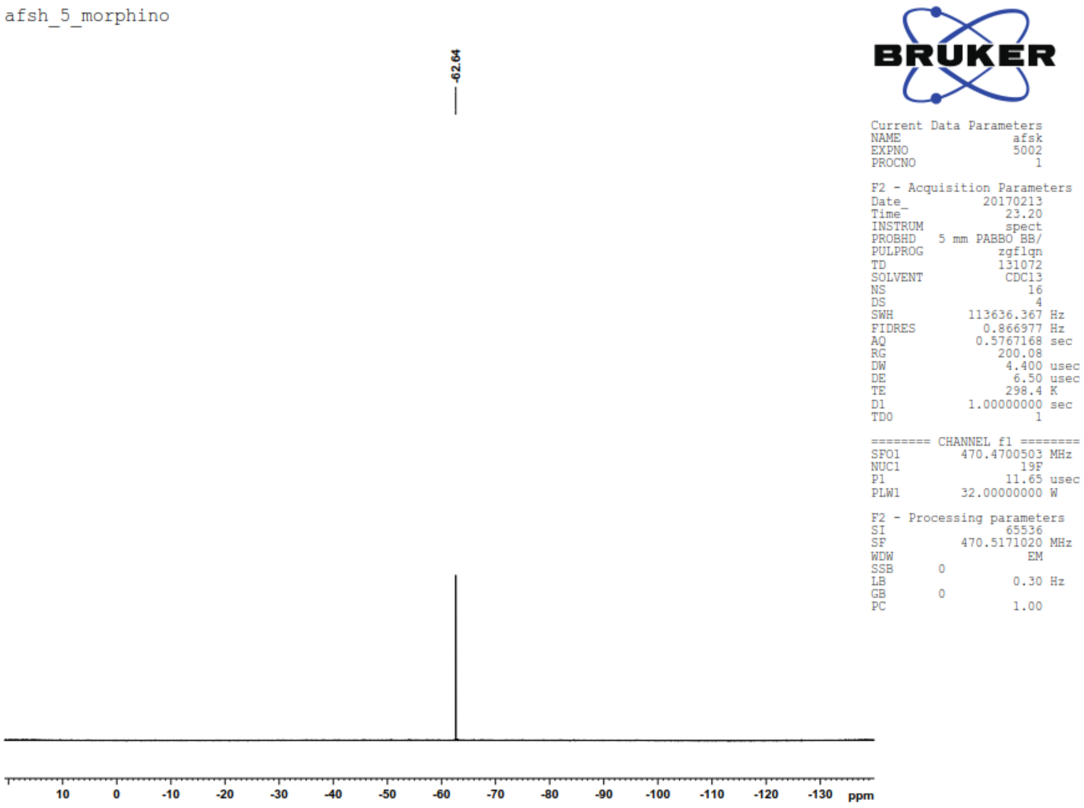

Figure S36: ^{19}F NMR spectra of compound 19

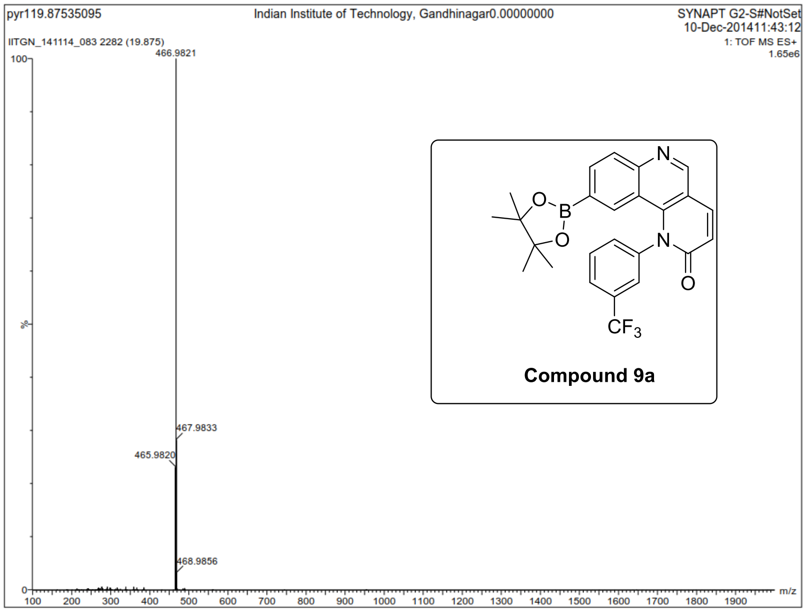

Figure S37: LC-Mass of compound 9a

Cell based studies

Materials and Methods

Colon cancer cell line, HCT-116, was received as a kind gift from Dr. Virupakshi Sopinna (IIT Gandhinagar). DMEM, fetal bovine serum, pen-strep and cell extraction buffer were purchased from Invitrogen Corporation (Carlsbad, CA, USA). Complete EDTA-Free protease inhibitor tablets were purchased from Roche (Basel, Switzerland). Sodium dodecyl sulfate, tetramethylenediamine (TEMED), ammonium per sulphate, Tween-20, β-mercatopethanol, bromophenol blue, non-fat milk and bovine serum albumin were purchased from Sigma-Aldrich (Darmstadt, Germany). Clarity ECL western blotting substrate and immunoblot PVDF western blotting membrane were purchased from Bio-Rad laboratories (Hercules, CA, USA). Rabbit anti-human Chk1 phospho Ser 317 (Cat. 12302), mouse anti-human phospho p70 S6 kinase Thr389 (Cat. 9206) were purchased from Cell Signaling Technology (MA, USA) and mouse anti-human β-actin (Cat. SC47778) was from Santa Cruz Biotechnology (Dallas, Texas, USA). Anti-rabbit IgG HRP-linked secondary antibody (Cat. 7074) and anti-mouse IgG HRP-linked secondary antibody (Cat. 7076) were obtained from Cell Signaling Technology (MA, USA). CellTiter-Glo® Luminescent cell viability assay kit was purchased from Promega Corporation (Madison, WI, USA). 96- and 6-well plates were purchased from Corning (New York, USA).

Cell viability assay

Human HCT-116 cells were grown in DMEM/10% FBS/1% Pen-Strep medium at 37^0C in a humidified incubator with 5% CO_2. For the cell viability assay, cells were plated into 96-well plates at a count of 2000 cells per well in 198 μl medium, incubated for 24 hours and then treated with increasing concentrations of

compound, respectively. After 72 hours of compound treatment, cell viability was determined using CellTiter-Glo® (Promega). Luminescence was measured by Envision Hybrid and modular multimode reader. All data were calculated by GraphPad Prism 6 software to get GI_{50} of each compound.

Immunoblot assay

HCT-116 cells were seeded in 6-well plates at a count of 0.5 x 10^6 cells per well and incubated overnight in a humidified CO_2 incubator maintained at 37^0C. For ATR assay, cells were exposed to 50 mJ/cm^2 of UV radiation energy (using UVP cross linker) after an hour of pre-treatment with appropriate compounds. Culture media was saved before UV treatment and added back to the cells after UV treatment. After another 1-hour incubation, cells were rinsed with ice-cold PBS and lysed in ice-cold cell extraction buffer. The soluble fractions of cell lysate were isolated by centrifugation at 13000rpm for 10 minutes at 4^0C. Following that, concentration of the protein was normalized by Bradford assay. Cell lysates were then subjected to SDS-PAGE and immunoblotting.

Discussion

The anticancer activity of all the synthesized compounds was performed against HCT-116 cell line at 1 μM concentration (Figure 7). Compound 13 and 14 showed strong inhibition similar to compound 11 (Torin2). Two novel potent compound inhibitors i.e., 13 and 14 were selected from the initial screening based cell viability assay and used to sensitize colon cancer cell line. We performed another cell viability assay across a dose range of both the compounds in colon cancer cell line. A dose range between 0nM and 1000nM was chosen. With an incubation time of up to 72 hours, compound 14 was more toxic than 13 with a GI_{50} of 57 nM (Figure S38). Compound 13 also inhibited viability of colon cancer cells with a GI_{50} of 138 nM (Figure S38). We confirmed that both 13 and 14 helped in sensitization of HCT-116 cells.

Based on the above mentioned results, to confirm if the compounds inhibit ATR and mTOR signaling in colon cancer cell line treated with radiation, an immunoblot assay was performed. Radiation was used as a part of combinatorial treatment as it is commonly used in colon cancer treatment. We assessed phosphorylation of Chk1 (p-$Chk1^{S317}$) and p70 S6 kinase (p70 $S6K^{T389}$) by immunoblotting of treated HCT-116 cells. p-$Chk1^{S317}$ and p70 $S6K^{T389}$ are downstream targets of ATR and mTOR, respectively. It was observed that 250 nM of 14 treatment inhibited phosphorylation of Chk1 (Ser 317) after treatment with UV radiation (Figure S40). Interestingly, phosphorylation of p70 S6 kinase (Thr 389) was observed to be inhibited by both compounds at 50 nM for 14 and 250 nM for 13 (Figure S40 and S41). Importantly, we confirmed that compound 14 inhibited the phosphorylation of ATR and mTOR substrates under these conditions. Whereas compound 13 inhibited the phosphorylation of mTOR substrate but not the ATR kinase substrate under UV treatment.

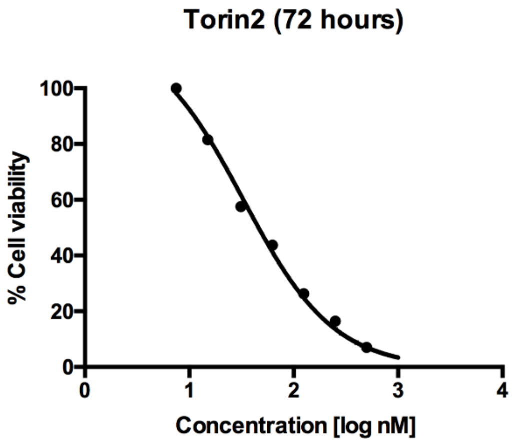

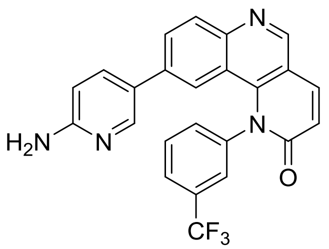

Torin2/11

GI_{50} = 32.88 nM

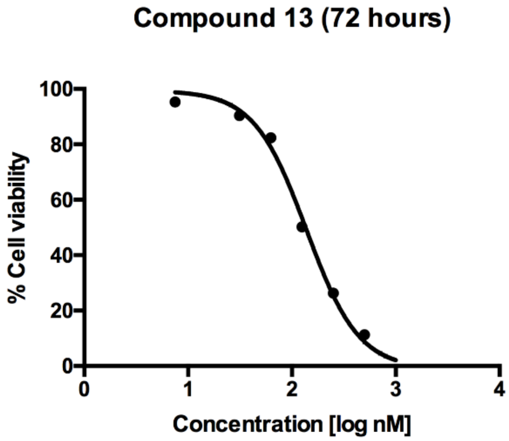

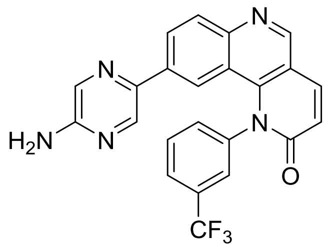

13

GI_{50} = 138 nM

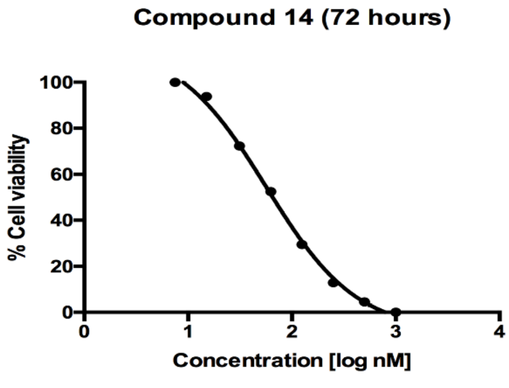

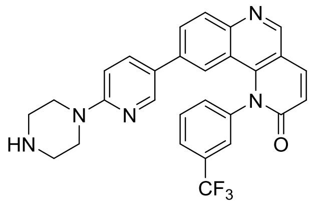

14

GI_{50} = 57 nM

Figure S38: HCT-116 cell line was treated with increasing concentration of compound 11 (Torin2), compound 13 and 14 to determine their GI_{50} values.

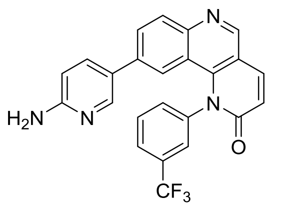

Torin2/11

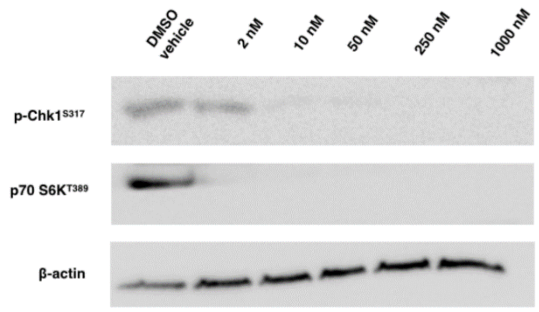

Figure S39: Torin2 /Compound 11 inhibits p-Chk1^{S317} and p70 S6K^{T389} phosphorylation in HCT-116 cell line (cells were irradiated with 50 mJ/cm^2 of UV radiation energy).

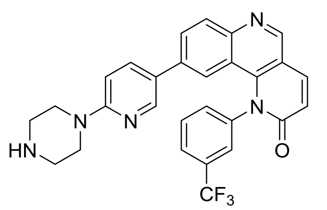

14

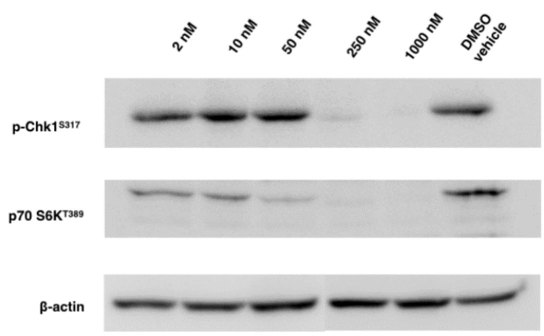

Figure S40: Compound 14 inhibits p-Chk1^{S317} and p70 S6K^{T389} phosphorylation in HCT-116 cell line (cells were irradiated with 50 mJ/cm^2 of UV radiation energy).

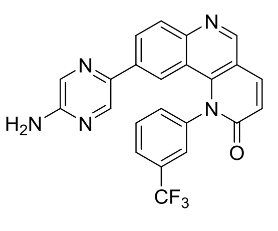

13

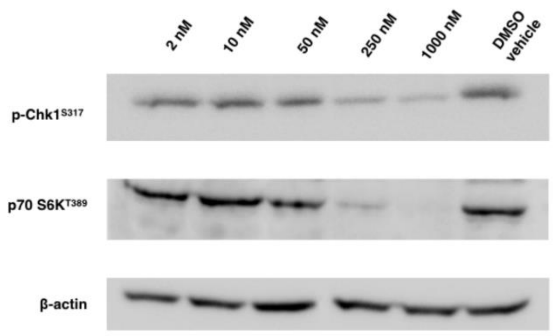

Figure S41: Compound 13 inhibits p70 S6K ^{T389} phosphorylation in HCT-116 cell line (cells were irradiated with 50 mJ/cm^2 of UV radiation energy).
